# A circular intronic RNA ciPVT1 delays endothelial cell senescence by regulating the miR‐24‐3p/CDK4/pRb axis

**DOI:** 10.1111/acel.13529

**Published:** 2021-12-13

**Authors:** Xue Min, Meng‐yun Cai, Tong Shao, Zi‐yang Xu, Zhaofu Liao, Dong‐liang Liu, Meng‐yuan Zhou, Wei‐peng Wu, Yu‐lan Zhou, Miao‐hua Mo, Shun Xu, Xinguang Liu, Xing‐dong Xiong

**Affiliations:** ^1^ Guangdong Provincial Key Laboratory of Medical Molecular Diagnostics Institute of Aging Research Guangdong Medical University Dongguan China; ^2^ Institute of Biochemistry & Molecular Biology Guangdong Medical University Zhanjiang China; ^3^ Clinical Research Center Affiliated Hospital of Guangdong Medical University Zhanjiang China

**Keywords:** CDK4, ciPVT1, endothelial cell senescence, miR‐24‐3p, pRb

## Abstract

Circular RNAs (circRNAs) have been established to be involved in numerous processes in the human genome, but their function in vascular aging remains largely unknown. In this study, we aimed to characterize and analyze the function of a circular intronic RNA, ciPVT1, in endothelial cell senescence. We observed significant downregulation of ciPVT1 in senescent endothelial cells. In proliferating endothelial cells, ciPVT1 knockdown induced a premature senescence‐like phenotype, inhibited proliferation, and led to an impairment in angiogenesis. An *in vivo* angiogenic plug assay revealed that ciPVT1 silencing significantly inhibited endothelial tube formation and decreased hemoglobin content. Conversely, overexpression of ciPVT1 in old endothelial cells delayed senescence, promoted proliferation, and increased angiogenic activity. Mechanistic studies revealed that ciPVT1 can sponge miR‐24‐3p to upregulate the expression of CDK4, resulting in enhanced Rb phosphorylation. Moreover, enforced expression of ciPVT1 reversed the senescence induction effect of miR‐24‐3p in endothelial cells. In summary, the present study reveals a pivotal role for ciPVT1 in regulating endothelial cell senescence and may have important implications in the search of strategies to counteract the development of age‐associated vascular pathologies.

## Graphical abstract

We characterized a novel circular intronic RNA ciPVT1, which originates from intron 4 of the PVT1 gene, was markedly reduced in senescent endothelial cells. Further functional and mechanistic investigations revealed that ciPVT1 may act as a competing endogenous RNA (ceRNA) to regulate CDK4 and its downstream gene by decoying miR‐24‐3p, thereby delaying endothelial cell senescence.

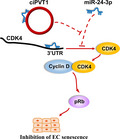

## 1 INTRODUCTION

Endothelial cells (ECs) line the inner surface of the blood vessels and form a protective barrier with multifunctional properties that include maintenance of vascular homeostasis, regulation of blood pressure, promotion of angiogenesis, and control of the coagulation process (Tesauro et al., 2017). Similar to other normal diploid cells, ECs have a finite cell lifespan and ultimately enter a state of replicative senescence (Foreman & Tang, 2003). Many stimuli such as oxidative stress and inflammation can also induce and promote cellular senescence in ECs (Donato et al., 2015). Endothelial cell senescence is associated with endothelial dysfunction which is an independent risk factor for the development of atherosclerosis and hypertension (Herrera et al., 2010). Therefore, clarifying the underlying mechanism of endothelial cell senescence may contribute to the development of approaches to prevent and treat age‐associated cardiovascular diseases.

Circular RNAs (circRNAs) comprise a large class of endogenous biomolecules that are produced by a non‐canonical splicing event called backsplicing. During backsplicing, a downstream splice‐donor site is covalently linked to an upstream splice‐acceptor site, resulting in the formation of exonic circRNAs or exon‐intron circRNAs (Barrett et al., 2015; Li et al., 2015; Zhang et al., 2014). Alternatively, circular intronic RNAs (ciRNAs), another class of circular RNA molecules, are generated from intronic lariat precursors that escape from the debranching step of canonical linear splicing (Zhang et al., 2013). In the past 40 years, circRNA was observed in the cytoplasm of eukaryotic cells by electron microscopy and considered as a by‐product of a splicing error (Hsu & Coca‐Prados, 1979). With the advent of high‐throughput RNA sequencing and circRNA‐specific bioinformatics algorithms, large numbers of circRNAs have been successively identified in a wide range of mammalian tissues. Some of them are abundant, stable, and specifically expressed in tissues, suggesting that they may have regulatory functions in the cell (Jeck & Sharpless, 2014; Memczak et al., 2013; Salzman et al., 2013; Wang et al., 2014). The known action modes of circRNAs are to serve as microRNA (miRNA) sponges, bind and sequester RNA‐binding proteins, modulate transcription and splicing, and encode peptides or proteins (Chen, 2016; Kristensen et al., 2019). Several lines of evidence indicate that circRNAs are involved in the regulation of various physiological and pathophysiological processes (Boeckel et al., 2015; Wang et al., 2016; Zheng et al., 2016). However, the role of circRNAs in endothelial cell senescence remains largely unknown.

In the current study, we identified and characterized a circular intronic RNA ciPVT1, which originates from intron 4 of the *PVT1* gene, was markedly reduced in senescent endothelial cells. Further functional and mechanistic investigations revealed that ciPVT1 may act as a competing endogenous RNA (ceRNA) to regulate CDK4 and its downstream gene by decoying miR‐24‐3p, thereby delaying endothelial cell senescence.

## 2 RESULTS

### 2.1 Identification and characteristics of ciPVT1 in ECs

**FIGURE 1:**
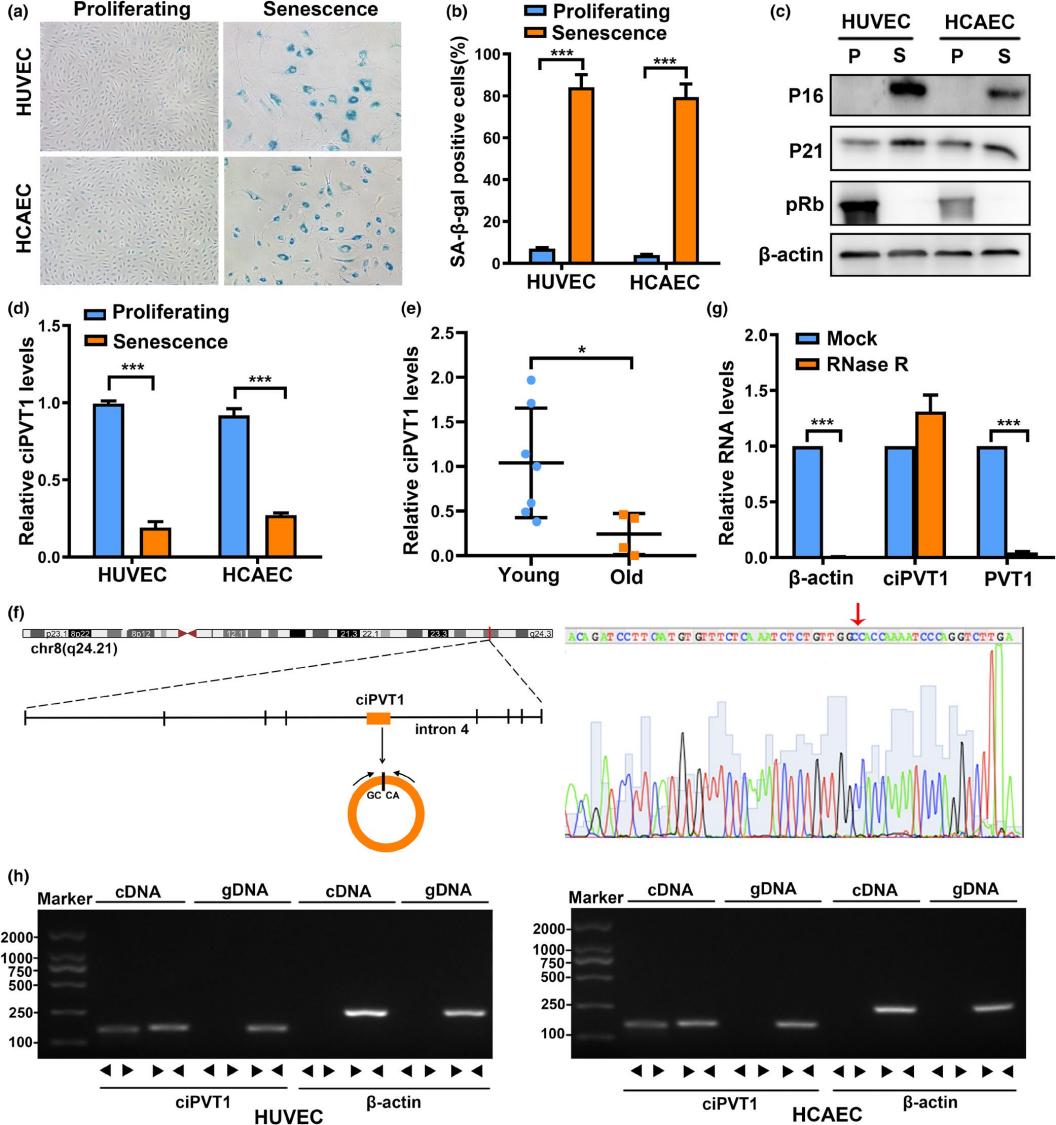
Validation and characteristics of ciPVT1 in ECs. (a) Micrographs to visualize SA‐β‐gal activity in proliferating and senescent ECs. (b) SA‐β‐gal staining positive cells were counted and presented as percentage of total cells. (c) Western blot analysis of the levels of P21, P16, pRb, and β‐actin in proliferating (P) and senescent (S) ECs. (d) RT‐qPCR analysis of ciPVT1 expression in proliferating and senescent ECs. (e) The expression levels of ciPVT1 were validated in blood vessels of young (*n* = 7) and old (*n* = 4) individuals using RT‐qPCR. (f) Scheme illustrating the production of ciPVT1. PCR primers used to specifically detect ciPVT1 by RT‐qPCR are indicated by black arrows. The presence of ciPVT1 was validated by RT‐PCR, followed by Sanger sequencing. Red arrow represents “head‐to‐tail” ciPVT1 splicing sites. (g) RT‐qPCR analysis of ciPVT1 and PVT1 RNA after treatment with RNase R in HUVECs. The relative levels of ciPVT1 and PVT1 RNA were normalized to the value measured in the mock treatment. (h) RT‐PCR or PCR assay indicating the detection of ciPVT1 using divergent and convergent primers from cDNA or genomic DNA (gDNA) of ECs. β‐actin was used as negative control. Data are presented as mean ± SD; *p < 0.05, ***p < 0.001

Replicative senescent ECs displayed a flattened and enlarged morphology, and increased SA‐β‐gal activity compared with proliferating ECs (Figure 1a,b). Western blot analysis revealed that senescent ECs expressed lower levels of pRb, while they expressed higher levels of P16 and P21 (Figure 1c). According to our previous RNA‐seq data (the dataset is available from Gene Expression Omnibus, GSE151475), ciPVT1 was markedly downregulated in both senescent HUVECs and HCAECs, suggesting that ciPVT1 may play a role in endothelial cell senescence. Then, the expression of ciPVT1 was validated to be in accordance with our RNA‐seq results by RT‐qPCR (Figure 1d). Additionally, the expression of ciPVT1 declined in ECs during continual passaging (Figure S1a,c). Consistent with our observation in ECs, ciPVT1 levels in human arteries were significantly lower in old individuals than those in young individuals (Figure 1e). Moreover, we further established another typical cellular senescence model, H_2_O_2_‐induced premature senescence of ECs. As shown in Figure S2a–c, H_2_O_2_ treatment significantly increased SA‐β‐gal‐positive cells and the protein levels of P53 and P21. Similarly, the expression levels of ciPVT1 also decreased in H_2_O_2_‐induced senescent ECs (Figure S2d), which further indicated the association of ciPVT1 with EC senescence.

CiPVT1 (circBase ID: hsa_circ_0008849) was derived from intron 4 of the *PVT1* gene (Figure 1f). We confirmed the head‐to‐tail splicing in the RT‐PCR product of ciPVT1 with expected size by Sanger sequencing (Figure 1f). Moreover, RT‐qPCR confirmed that ciPVT1 was resistant to RNase R, while PVT1 RNA level was significantly reduced after RNase R treatment (Figure 1g). Using cDNA and genomic DNA (gDNA) from HUVECs and HCAECs as templates, ciPVT1 was only amplified by divergent primers in cDNA, and no amplification product was observed in gDNA (Figure 1h). Collectively, these findings demonstrated that ciPVT1 is a circular and stable transcript, significantly downregulated in senescent ECs.

### 2.2 CiPVT1 delayed senescence, induced proliferation, and increased angiogenic activity of ECs

**FIGURE 2:**
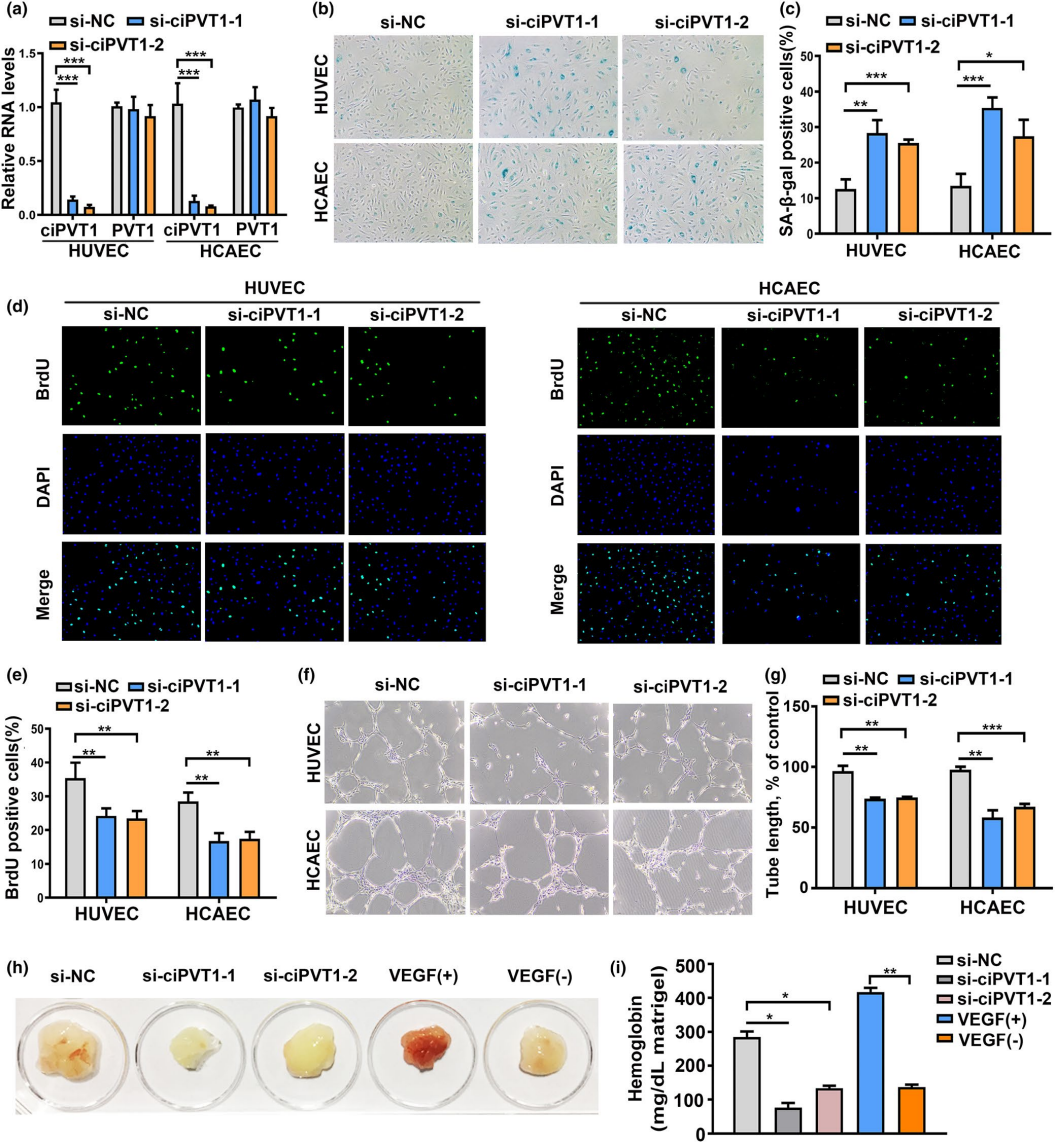
Knockdown of ciPVT1 promoted senescence, inhibited proliferation, and angiogenesis of ECs. (a) RT‐qPCR analysis of ciPVT1 and PVT1 RNA expression in proliferating ECs 3 days after transfection with si‐NC, si‐ciPVT1‐1, and si‐ciPVT1‐2. (b) Representative photographs of SA‐β‐gal staining of ECs transfected with si‐NC, si‐ciPVT1‐1, and si‐ciPVT1‐2. (c) The SA‐β‐gal‐positive cells were counted and presented as percentage of total cells. (d) Representative images of the indicated cells stained with DAPI (blue fluorescence) and BrdU, as a measurement of DNA synthesis (green fluorescence) in si‐NC‐, si‐ciPVT1‐1‐, and si‐ciPVT1‐2‐transfected ECs. (e) The BrdU‐positive cells were counted and presented as percentage of total cells. (f and g) Representative micrographs and statistical summary of *in vitro* Matrigel assays in si‐NC‐, si‐ciPVT1‐1‐, and si‐ciPVT1‐2‐transfected ECs. (h) CiPVT1 and angiogenesis *in vivo*. Athymic nude mice received a subcutaneous injection of Matrigel plugs supplemented with saline, VEGF, or mixed with HUVECs transfected with si‐NC, si‐ciPVT1‐1, and si‐ciPVT1‐2. Representative gross appearance of Matrigel plugs. (i) Quantification of hemoglobin (Hb) in the homogenized Matrigel plugs. Data are presented as mean ± SD; *p < 0.05, **p < 0.01, ***p < 0.001

To investigate the role of ciPVT1 in endothelial cell senescence, we designed two small interfering RNAs (siRNAs) targeting the back‐splice sequence. As expected, the siRNAs directed against the back‐splice sequence inhibited only the circular transcript of ciPVT1 but did not affect the expression of the PVT1 linear species (Figure 2a). Silencing ciPVT1 triggered an enlarged and flattened cell morphology accompanied by increased SA‐β‐gal activity (Figure 2b,c). There is also evidence shown that senescent endothelial cells do not proliferate and that their angiogenic capacity is impaired (Ungvari et al., 2018). As shown in Figure 2d–g, reduced ciPVT1 expression inhibited proliferation and impinged on the ability of ECs to form capillary‐like networks. Moreover, the *in vivo* angiogenic activity of endothelial cells was investigated using the Matrigel plug assay. We found that the plugs mixed with si‐ciPVT1‐transfected HUVECs showed less new vessel formation macroscopically and had lower hemoglobin content in the Matrigel plugs than the si‐negative control (si‐NC) group (Figure 2h,i). H&E and CD31 immunofluorescence staining also revealed obviously less numbers of vessel or capillary‐like structures in the Matrigel plugs of the si‐ciPVT1 groups (Figure S3). In addition, cell cycle analysis illustrated that silencing of ciPVT1 decreased the number of cells in S phase, but increased the number of cells in G1 phase as compared with the controls (Figure S4). Next, ECs were infected with the ciPVT1 lentivirus and, as a result, the expression of ciPVT1, but not PVT1 RNA, significantly increased (Figure S5a). Overexpression of ciPVT1 delayed senescence, promoted proliferation, and increased angiogenic activity of ECs (Figure S5b–g). Taken together, these results imply the involvement of ciPVT1 in the regulation of the endothelial cell senescent phenotype.

### 2.3 CiPVT1 may function as a sponge for miR‐24‐3p

**FIGURE 3:**
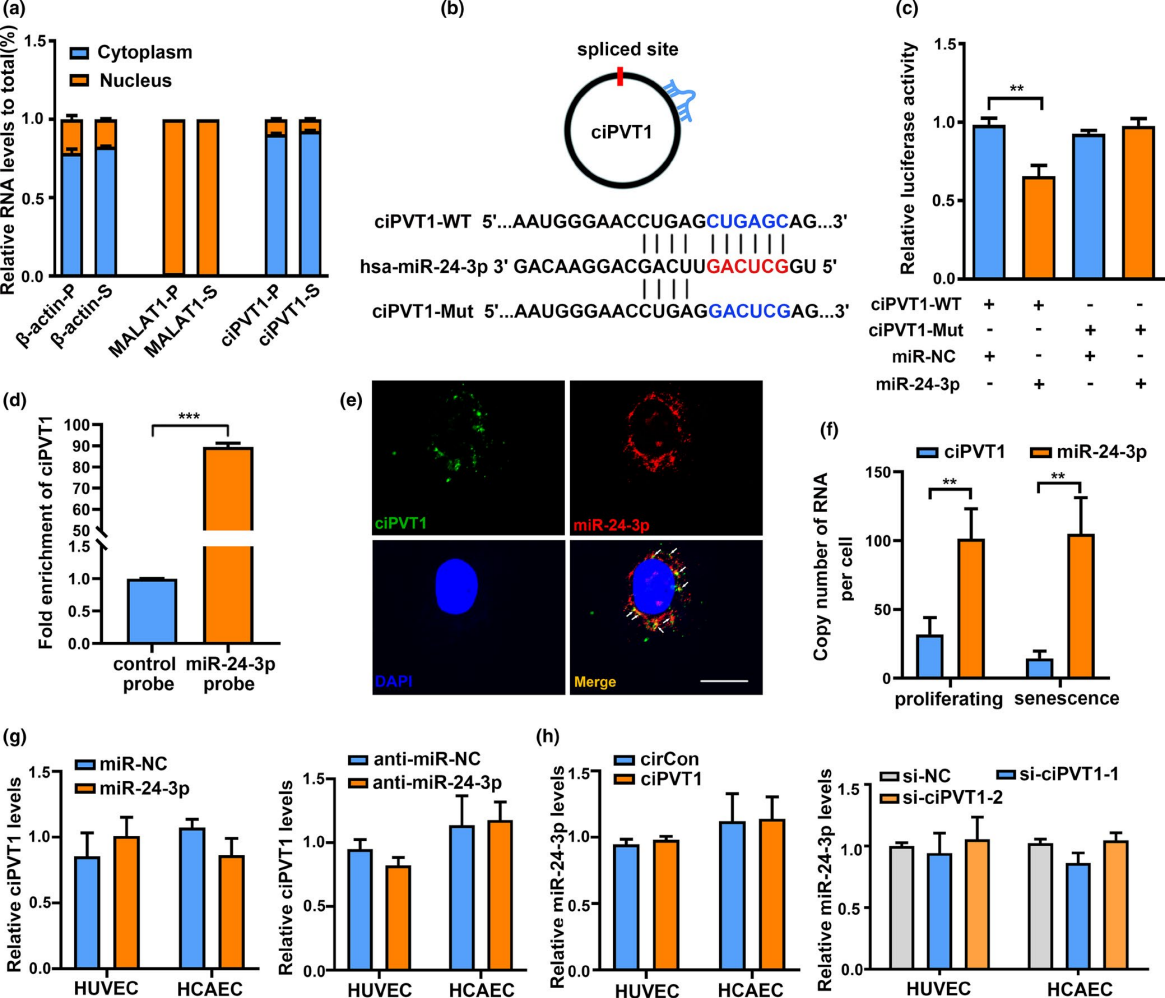
CiPVT1 functioned as a sponge for miR‐24‐3p. (a) RT‐qPCR analysis of ciPVT1 in either the cytoplasm or nucleus in proliferating (P) and senescent (S) HUVECs. β‐actin and MALAT1 were applied as positive controls in the cytoplasm and nucleus, respectively. (b) Schematic illustration of ciPVT1‐WT and ciPVT1‐Mut luciferase reporter vectors. (c) Relative luciferase activities were measured in 293T cells after transfection with ciPVT1‐WT or ciPVT1‐Mut and miR‐NC or miR‐24‐3p, respectively. (d) RT‐qPCR was used to detect the expression of ciPVT1 in the miR‐24‐3p pull‐down complex in HUVECs. (e) Fluorescence in situ hybridization (FISH) showing co‐localization between ciPVT1 and miR‐24‐3p in HUVECs. CiPVT1 probes were labeled with FAM. MiR‐24‐3p probes were labeled with Cy3. Nuclei were stained with DAPI. Scale bar, 20 μm. (f) The copy numbers of ciPVT1 and miR‐24‐3p in proliferating and senescent HUVECs were determined by absolute quantification. (g) ECs were transfected with miR‐24‐3p or miR‐NC, and anti‐miR‐24‐3p or anti‐miR‐NC. RT‐qPCRs were conducted to detect ciPVT1 expression. (h) ECs were infected with the circControl (circCon) or ciPVT1‐GFP lentivirus, or transfected with si‐NC, si‐ciPVT1‐1, and si‐ciPVT1‐2. RT‐qPCRs were conducted to detect miR‐24‐3p expression. Data are presented as mean ± SD; **p < 0.01, ***p < 0.001

Growing evidence has shown that circRNAs might play different roles based on their subcellular localization (Zhou et al., 2018). To determine the cellular localization of ciPVT1, RT‐qPCR analysis was conducted for nuclear and cytoplasmic ciPVT1 RNA. Results showed that ciPVT1 was preferentially localized within the cytoplasm in both proliferating and senescent endothelial cells, suggesting its post‐transcriptional regulatory potential (Figure 3a). Given that circRNAs have been reported to function as sponges for miRNAs and that ciPVT1 is stable and primarily located in the cytoplasm, we investigated whether ciPVT1 could bind to miRNAs. circRIP was performed to identify ciPVT1‐associated miRNAs using a biotin‐labeled probe specifically against ciPVT1. We found a specific enrichment of ciPVT1 and several miRNAs compared with the control in ciPVT1‐overexpressing stable cells (Table S1). Among them, miR‐24‐3p showed the highest enrichment in the circRIP assay (Table S1). A potential binding site of miR‐24‐3p was found within the ciPVT1 sequence using RNAhybrid (Figure 3b). To validate the bioinformatics prediction, a dual‐luciferase reporter assay was applied in 293T cells. The full length of ciPVT1‐wild type (WT) and mutant version without the miR‐24‐3p binding site were subcloned into the luciferase reporter vector pEZX‐GA02 (Figure 3b). The results showed that miR‐24‐3p could significantly decrease the luciferase activity of the WT group, but not the mutant one (Figure 3c), suggesting that there might be a direct interaction between ciPVT1 sequence and miR‐24‐3p. We then used a biotin‐labeled miR‐24‐3p probe to pull‐down circRNAs and observed obvious enrichment of ciPVT1 compared with the control probe (Figure 3d). Moreover, RNA FISH assay revealed that ciPVT1 and miR‐24‐3p were co‐localized in the cytoplasm (Figure 3e). We quantified the absolute copy number of ciPVT1 and miR‐24‐3p in HUVECs (Figure 3f). Results showed that the observed ciPVT1/miR‐24‐3p ratio seemed to be compatible with a sponge activity. Furthermore, ciPVT1 did not show significant changes after overexpression or inhibition of miR‐24‐3p (Figure 3g), and overexpressing or silencing of ciPVT1 had no impact on the expression of miR‐24‐3p (Figure 3h), suggesting that ciPVT1 and miR‐24‐3p may not be digested by each other. Collectively, the above results demonstrate that ciPVT1 functions as a sponge for miR‐24‐3p.

### 2.4 MiR‐24‐3p promoted senescence, reduced proliferation, and impaired the capillary tube network formation ability of ECs

**FIGURE 4:**
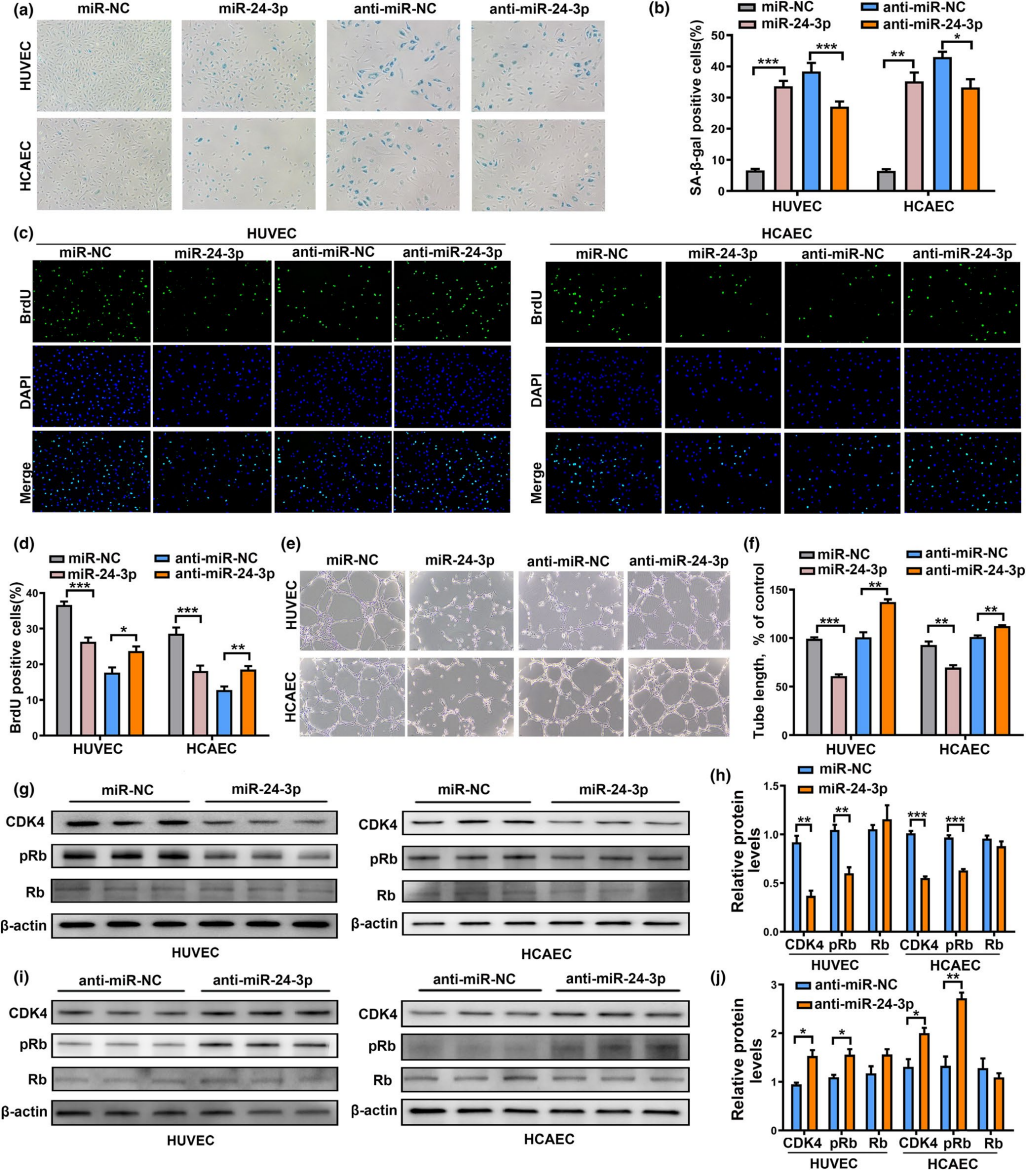
MiR‐24‐3p promoted senescence, reduced proliferation, and impaired the capillary tube network formation ability of ECs. (a) Representative photographs of SA‐β‐gal staining of ECs transfected with miR‐24‐3p or miR‐NC, and anti‐miR‐24‐3p or anti‐miR‐NC. (b) The number of senescent cells was counted and presented as percentage of SA‐β‐gal‐positive cells. (c) Representative images of cells stained with DAPI and BrdU in miR‐24‐3p‐ or miR‐NC‐, and anti‐miR‐24‐3p‐ or anti‐miR‐NC‐transfected ECs. (d) The BrdU‐positive cells were counted and presented as percentage of total cells. (e and f) Representative micrographs and statistical summary of *in vitro* Matrigel assays in miR‐24‐3p‐ or miR‐NC‐, and anti‐miR‐24‐3p‐ or anti‐miR‐NC‐transfected ECs. (g and h) CDK4, pRb, Rb protein expression, and intensity ratio between CDK4, pRb, Rb, and β‐actin in ECs transfected with miR‐24‐3p or miR‐NC. (i and j) CDK4, pRb, Rb protein expression, and intensity ratio between CDK4, pRb, Rb, and β‐actin in ECs transfected with anti‐miR‐24‐3p or anti‐miR‐NC. Data are presented as mean ± SD; *p < 0.05, **p < 0.01, ***p < 0.001

It has been reported that miR‐24 is upregulated in senescent HDFs (human diploid fibroblasts) and HUVECs, suggesting its role in cellular senescence (Dellago et al., 2013; Khee et al., 2014). To investigate the function of miR‐24‐3p in endothelial cell senescence, HUVECs and HCAECs were transfected with miR‐24‐3p or miR‐NC and anti‐miR‐24‐3p or anti‐miR‐NC. Overexpression of miR‐24‐3p increased the number of SA‐β‐gal‐positive cells, inhibited cell proliferation, and impaired the tube formation ability of proliferating ECs (Figure 4a–f). In contrast, transfection of anti‐miR‐24‐3p showed opposite effects in senescent ECs (Figure 4a–f). These results confirm the crucial role of miR‐24‐3p in endothelial cell senescence.

A previous study has confirmed that CDK4 is a direct miR‐24‐3p target (Lal et al., 2009). CDK4 is a key regulator of the transition through the G1 phase of the cell cycle. The D‐type cyclin/CDK4 complex phosphorylates and inactivates the retinoblastoma (Rb) protein which is important for establishing senescent cell cycle arrest (Rodier & Campisi, 2011). Western blot assays demonstrated that the transfection of miR‐24‐3p mimics reduced endogenous CDK4 and phosphorylated Rb (pRb) protein levels in ECs (Figure 4g,h). Anti‐miR‐24‐3p increased CDK4 and pRb protein levels in ECs (Figure 4i,j). However, the expression levels of Rb were not altered by miR‐24‐3p overexpression or inhibition (Figure 4g–j). These results reveal that miR‐24‐3p can induce senescence, inhibit cell proliferation, and impair the tube formation ability of ECs via the CDK4/pRb pathway.

### 2.5 Overexpression of ciPVT1 alleviates miR‐24‐3p‐induced cellular senescence in ECs

**FIGURE 5:**
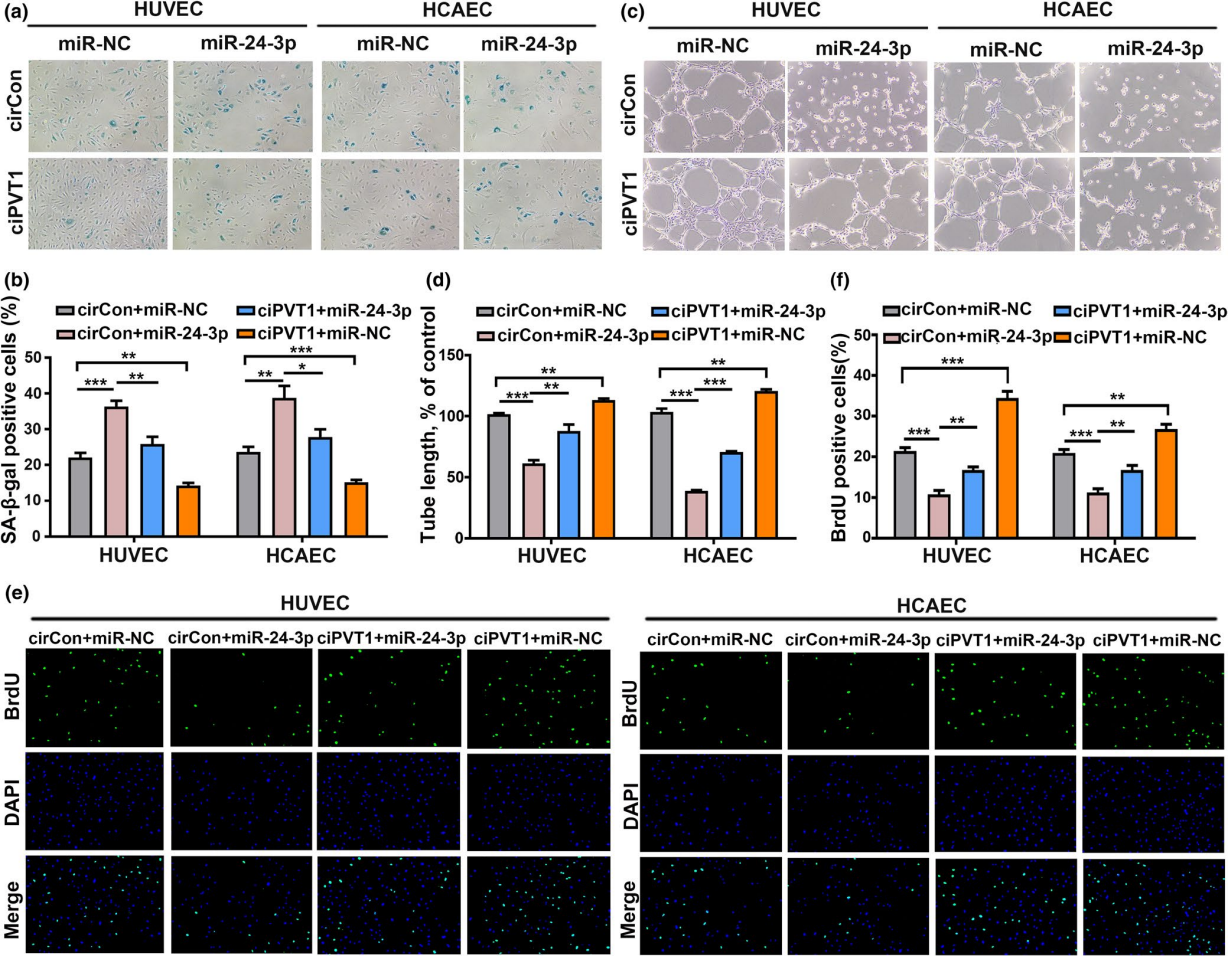
Overexpression of ciPVT1 alleviated miR‐24‐3p‐induced cellular senescence in ECs. SA‐β‐gal staining (a and b), *in vitro* Matrigel assays (c and d), and BrdU (e and f) showed that miR‐24‐3p could promote endothelial cell senescence, impair the tube formation ability and inhibit cell proliferation of ECs. Overexpression of ciPVT1 could rescue these phenotypes induced by miR‐24‐3p overexpression. Data are presented as mean ± SD; *p < 0.05, **p < 0.01, ***p < 0.001

Next, we examined whether ciPVT1 delayed senescence, promoted cell proliferation, and increased angiogenic activity via interacting with miR‐24‐3p. SA‐β‐gal staining showed that miR‐24‐3p could promote endothelial cell senescence and that the effect could be alleviated by overexpression of ciPVT1 (Figure 5a,b). MiR‐24‐3p could impair the tube formation ability and inhibit cell proliferation of ECs. Likewise, overexpression of ciPVT1 could rescue these phenotypes induced by miR‐24‐3p overexpression (Figure 5c–f). Together, these data suggest that ciPVT1 delayed endothelial cell senescence via sponging miR‐24‐3p.

### 2.6 CiPVT1 delayed endothelial cell senescence by regulating the miR‐24‐3p/CDK4/pRb axis

**FIGURE 6:**
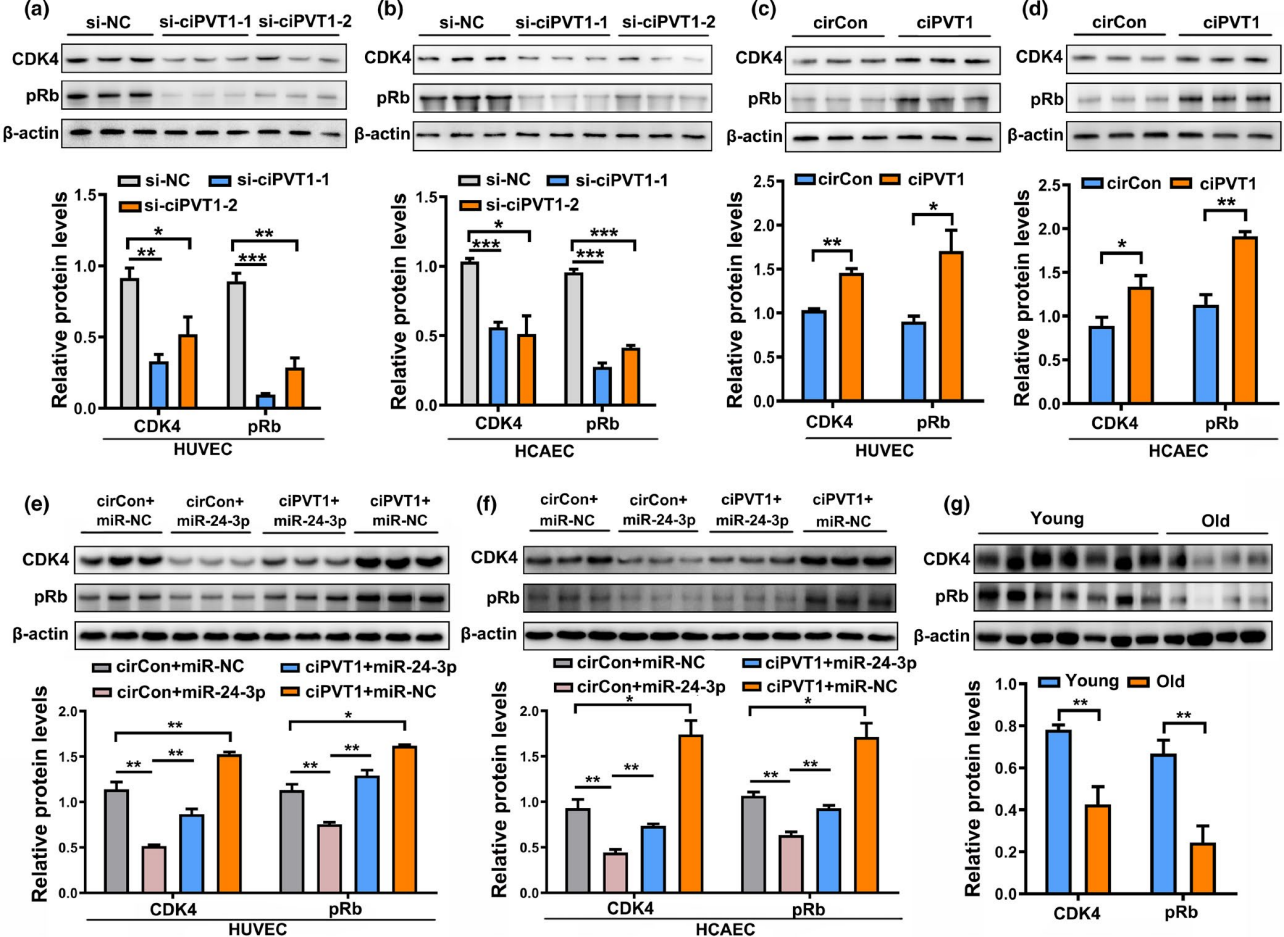
CiPVT1 delayed endothelial cell senescence by regulating the miR‐24‐3p/CDK4/pRb axis. (a and b) Western blot analysis of the levels of CDK4 and pRb in HUVECs and HCAECs transfected with si‐NC, si‐ciPVT1‐1, and si‐ciPVT1‐2. (c and d) Western blot analysis of the levels of CDK4 and pRb in HUVECs and HCAECs transfected with circControl (circCon) or ciPVT1‐GFP lentivirus. (e and f) MiR‐24‐3p could significantly suppress the expression of CDK4 and pRb, and the suppression was retarded after overexpression of ciPVT1 in HUVECs and HCAECs. (g) CDK4, pRb protein expression in blood vessels of young (*n* = 7) and old (*n* = 4) individuals. Data are presented as mean ± SD; *p < 0.05, **p < 0.01, ***p < 0.001

As miR‐24‐3p facilitates endothelial cell senescence through targeting CDK4 (Figure 4), we hypothesized that ciPVT1 competed by binding to miR‐24‐3p and increased the levels of CDK4/pRb, thus delaying the senescence of ECs. We detected the expression of CDK4/pRb after overexpressing or silencing ciPVT1. The results showed that CDK4/pRb was upregulated when ciPVT1 was overexpressed, and was downregulated when ciPVT1 was knocked down (Figure 6a–d). This effect may be caused by the ability of ciPVT1 to bind to the inhibitor of CDK4, miR‐24‐3p, and thus upregulate CDK4/pRb more efficiently. Next, we determined whether ciPVT1 delays the senescence of ECs mainly by protecting CDK4 from downregulation by miR‐24‐3p. As expected, we observed that exogenous miR‐24‐3p could significantly suppress the expression of CDK4 and that the suppression was retarded after overexpression of ciPVT1 (Figure 6e,f). We then mutated miR‐24‐3p‐binding site on ciPVT1 (ciPVT1‐miR‐mut). As shown in Figure S6, overexpression of a mutant ciPVT1 lacking the ability to bind miR‐24‐3p had no effect on cellular senescence, proliferation, angiogenic activity of ECs and the levels of CDK4/pRb, suggesting that ciPVT1 servers as a sponge for miR‐24‐3p to regulate CDK4 via the ceRNA mechanism in ECs. Consistent with the expression of ciPVT1 (Figure 1e), the expression levels of CDK4 and pRb were also downregulated in human arteries of old individuals (Figure 6g). These results confirmed that a reciprocal association exists *in vivo* between the expression levels of ciPVT1 and CDK4/pRb. Collectively, these observations demonstrate that ciPVT1 delayed senescence, induced proliferation, and increased angiogenic activity of ECs, at least partly, through the miR‐24‐3p/CDK4/pRb pathway.

## 3 DISCUSSION

Cellular senescence is characterized by permanent exit from the cell cycle and the appearance of distinct morphological and functional changes associated with an impairment of cellular homeostasis (Campisi & d'Adda di Fagagna, 2007). In endothelial cells, the loss of replicative capacity resulting from the senescent state militates against the integrity of the endothelium and impairs successful angiogenesis. Furthermore, the endothelial senescent phenotype is pro‐inflammatory, pro‐atherosclerotic, and pro‐thrombotic (Erusalimsky, 2009). Thus, endothelial cell senescence promotes endothelial dysfunction and may contribute to the pathogenesis of age‐associated vascular disorders. Studying the molecular mechanism underlying endothelial cell senescence may provide new insights into managing vascular diseases. CircRNAs have recently gained attention because of their roles in gene expression regulation and various cellular processes (Barrett & Salzman, 2016). However, their function in endothelial cell senescence remains unknown. In this study, we revealed a novel circRNA termed ciPVT1, which was obviously diminished during endothelial cell aging. Gain‐of‐function and loss‐of‐function studies demonstrated that ciPVT1 could suppress senescence and increase angiogenic activity in ECs. These findings suggest that ciPVT1 is an essential regulator of endothelial cell senescence.

The ceRNA hypothesis states that RNA transcripts containing the same miRNA response elements can competitively inhibit the function of miRNAs, acting as RNA sponges to block and inhibit miRNAs from binding to their target sites (Salmena et al., 2011). Growing evidence indicated that circRNAs could serve as sponges for miRNAs to regulate the expression of miRNA target genes (Ren et al., 2020). The initial and seminal functional report on circRNA acting as a miRNA sponge is CDR1as, which contains over 70 binding sites for miR‐7 (Memczak et al., 2013). Besides, circTP63 competitively binds to miR‐873‐3p and prevents it from decreasing the level of FOXM1, which upregulates CENPA and CENPB, and finally facilitates cell cycle progression (Cheng et al., 2019). Moreover, circ‐Sirt1, as a miRNA sponge for miR‐132/212, promotes host gene SIRT1 expression in vascular smooth muscle cells (Kong et al., 2019). In our study, we investigated miRNAs which might interact with ciPVT1 and found that miR‐24‐3p showed the highest enrichment in ciPVT1 circRIP assays. Then, we compared the sequence of ciPVT1 with that of miR‐24‐3p using the bioinformatics program RNAhybrid and noticed that ciPVT1 contains a target site of miR‐24‐3p. Dual‐luciferase reporter, RNA pull‐down and FISH assays further confirmed that ciPVT1 could directly interact with miR‐24‐3p. Studies have revealed that miR‐24 is upregulated in senescent HDFs and HUVECs (Dellago et al., 2013; Khee et al., 2014). In this study, we found that miR‐24‐3p triggered endothelial cell senescence through targeting CDK4, resulting in downregulation of pRb. Further rescue experiments showed that ciPVT1 delayed endothelial cell senescence through the miR‐24‐3p/CDK4/pRb pathway. Our finding expands the understanding of the underlying mechanism of endothelial cell senescence.

CiPVT1 is an 855‐nt long circRNA which generated through backsplicing of intron 4 of the gene that encodes for long non‐coding RNA PVT1 (lncRNA‐NR_003367). Previous studies have suggested that *PVT1*‐encoded lncRNA or miRNAs have oncogenic functions (Onagoruwa et al., 2020). In this study, we found no significant change of lncRNA PVT1 expression in senescent ECs (Figure S1b,d) or H_2_O_2_‐induced senescent ECs (Figure S2d). Twenty‐six circular PVT1 isoforms have been annotated in the CircInteractome Database (https://circinteractome.nia. nih.gov/index.html) (Dudekula et al., 2016), the most common isoform is a 410‐nt exonic circRNA circPVT1 (hsa_circ_0001821). This 410‐nt circRNA was first characterized as a new proliferative factor and prognostic marker in gastric cancer (Chen et al., 2017). CircPVT1 is regulated through the mutant‐p53/YAP/TEAD complex via its regulatory region and behaves as an oncogene in head and neck squamous cell carcinoma (Verduci et al., 2017). Interestingly, circPVT1 is also a senescence‐associated circRNA exhibiting markedly reduced levels in senescent fibroblasts (Panda et al., 2017). However, we found that circPVT1 levels did not change significantly in senescent ECs relative to proliferating ECs (Figure S7). Thus, our data indicate that the circular intronic RNA ciPVT1, but not lncRNA PVT1 or circPVT1, is associated with endothelial cell senescence.

In conclusion, we characterized and functionally analyzed a ciRNA derived from intron 4 of the *PVT1* gene. We demonstrated that ciPVT1 is downregulated in senescent endothelial cells. Mechanistically, ciPVT1 delays endothelial cell senescence through targeting the miR‐24‐3p/CDK4/pRb pathway. Therefore, ciPVT1 could potentially be used as a promising target to prevent endothelial cell senescence and its related vascular complications.

## 4 MATERIALS AND METHODS

### 4.1 Cell culture and senescence induction

Human umbilical vein endothelial cells (HUVECs) and human coronary artery endothelial cells (HCAECs) were purchased from ScienCell Research Laboratories. The cells were cultured in endothelial cell medium supplemented with 5% fetal bovine serum and 1% endothelial cell growth supplement at 37°C in a humidified atmosphere of 5% CO_2_.

Replicative senescence model was established by serial passaging of ECs. HUVECs used at PDL <8 are referred to as “proliferating” cells, and PDL >29 as “senescent” cells. HCAECs used at PDL <9 are referred to as “proliferating” cells, and PDL >24 as “senescent” cells. The number of population doublings (PD) was calculated according to the equation PD = log_2_F/log_2_I (F, final population number; I, initial population number). The population‐doubling levels (PDLs) were calculated using the following formula: PDL(end) = PDL(initial) + PD. For H_2_O_2_‐induced senescence, proliferating ECs were treated with 100 μmol/L H_2_O_2_ for 1 h and incubated in complete medium for 7 days.

### 4.2 RNA isolation, RT‐qPCR analysis, and RNase R treatment

Total RNA was extracted from endothelial cells using TRIzol reagent (Thermo Fisher Scientific) according to the manufacturer's protocol. For miRNA, total RNA was reverse transcribed using the All‐in‐One miRNA RT‐qPCR Detection Kit (GeneCopoeia) according to the manufacturer's recommendations. RT‐qPCR was carried out in a LightCycler 96 System (Roche) with All‐in‐One miRNA RT‐qPCR Detection Kit (GeneCopoeia). Primers specific for miR‐24‐3p and U6 were all obtained from GeneCopoeia. For circRNA and mRNA, total RNA was reverse transcribed using the PrimeScript RT Reagent Kit with gDNA Eraser (Takara Bio), and RT‐qPCR was performed in a LightCycler 96 System with TB Green Premix Ex Taq II (Takara Bio). CircRNA was amplified using divergent primers to target the splice junction. β‐actin was used as internal reference for quantification of circRNA and mRNA, while U6 for miRNA. The specific primers used are listed in Table S2. The relative expression of genes was calculated using the 2^−ΔΔCT^ method. For RNase R treatment, 2 μg of total RNA was incubated for 15 min at 37°C with or without 5 U/μg RNase R (Lucigen), and then analyzed by RT‐qPCR.

### 4.3 Cell transfection

SiRNA targeting ciPVT1 and non‐specific negative control were purchased from RiboBio. The miRNA mimics/inhibitor and corresponding negative control for miR‐24‐3p were synthesized by GenePharma. The sequences used are listed in Table S2. Transfection was carried out using Lipofectamine RNAiMax Reagent (Thermo Fisher Scientific) according to the manufacturer's instructions.

### 4.4 Infection of ECs with lentivirus

Endothelial cells were infected with circControl (circCon), ciPVT1, or ciPVT1‐miR‐mut lentivirus (Geneseed) at a multiplicity of infection of 10, followed by gentle swirling, incubation, and replacement of fresh feed medium.

### 4.5 Senescence‐associated β‐galactosidase (SA‐β‐gal) staining

The Senescence β‐Galactosidase Staining Kit (Beyotime, Shanghai, China) was used for SA‐β‐gal staining according to the manufacturer's instructions. Briefly, cells were washed with PBS and fixed in SA‐β‐gal fixing solution for 15 min at room temperature. Subsequently, cells were washed thrice with PBS and stained with working solution at 37°C overnight. The images were taken at 100× magnification using an Eclipse TS100 Inverted Microscope (Nikon Corporation). The percentage of positively stained cells was determined by counting the cells in six randomly chosen microscopic fields.

### 4.6 BrdU incorporation assay

BrdU (5‐bromo‐2‐deoxyuridine) incorporation assay was performed to evaluate the capability of cell proliferation. Briefly, cells were incubated for 1 h with 40 μM BrdU in normal medium at 37°C and then fixed with 4% paraformaldehyde. The fixed cells were treated with 0.05% trypsin to permeabilize them, followed by incubation with 3% BSA for 1 h at room temperature or overnight at 4°C. Cells were further incubated with a mouse anti‐BrdU monoclonal antibody (Cell Signaling Technology), followed by an Alexa Fluor 488‐conjugated secondary antibody (Cell Signaling Technology). Finally, cells were stained with DAPI (Sigma‐Aldrich) as a counterstain, and images were captured at 100× magnification under an Invitrogen EVOS FL Auto Cell Imaging System (Thermo Fisher Scientific).

### 4.7 *In* *vitro* angiogenesis assay

For tube formation assays, HUVECs and HCAECs were seeded at a density of 3 × 10^4^ on 48‐well plates coated with 150 μl Matrigel (BD Biosciences). After culturing for 4–6 h, the cells were imaged at 100× magnification using an Eclipse TS100 Inverted Microscope and analyzed using the Image J programme. Tube length was determined by drawing a line along each tube and measuring the line in pixels.

### 4.8 Luciferase reporter assay

RNAhybrid was used to predict the potential binding site of miR‐24‐3p within the ciPVT1 sequence. The sequences of ciPVT1 and its corresponding mutant version without the miR‐24‐3p binding site, termed ciPVT1‐WT or ciPVT1‐Mut, respectively, were synthesized and subcloned into the luciferase reporter vector pEZX‐GA02 (GeneCopoeia). All constructed plasmids were validated by sequencing. Luciferase reporter vectors were co‐transfected with miR‐24‐3p or miR‐NC into 293T cells using Lipofectamine 3000 Reagent (Thermo Fisher Scientific). The relative luciferase activity was measured using the LucPair Duo‐Luciferase Assay Kit (GeneCopoeia) according to the manufacturer's instructions.

### 4.9 RNA pull‐down assay

The biotinylated miR‐24‐3p probes (5′ UGGCUCAGUUCAGCAGGAACAG ‐Biotin 3′) and the control probe (5′ UUCUCCGAACGUGUCACGU‐Biotin 3′) were synthesized by CLOUD‐SEQ Company (Shanghai, China). 1 × 10^7^ HUVECs were harvested, incubated in lysis buffer on ice for 10 min and the supernatant was collected by centrifugation. The probes were incubated with streptavidin‐coated magnetic beads to generate probe‐coated magnetic beads. The cell lysates were incubated with probe‐coated beads at 4°C overnight. After washing with the wash buffer, the RNA complexes bound to the beads were eluted and extracted using TRIzol. The abundance of ciPVT1 in the pull‐down materials was evaluated by RT‐qPCR analysis.

### 4.10 RNA fluorescence in situ hybridization (FISH)

Fluorescence in situ hybridization assay was executed to observe the location of ciPVT1 and miR‐24‐3p in HUVECs. Cells were hybridized with specific FAM‐labeled ciPVT1 probes and Cy3‐labeled miR‐24‐3p probes (Geneseed, Guangzhou, China) at 37°C overnight, and dyed with DAPI. The probes sequences were listed in Table S2. The images were acquired using an A1Si Laser Scanning Confocal Microscope (Nikon Instruments).

### 4.11 Digital PCR analysis

Absolute RNA quantitation was performed using a chip‐based dPCR platform (QuantStudioTM 3D Digital PCR System; Life Technologies). 2 μg of total RNA for probe qPCR assay was used in a 20 μl reverse‐transcription reaction. The final 14.5 μl of TaqMan PCR reaction mixture was made up in the following way: 7.25 μl QS3D digital PCR Master Mix v2, 0.725 μl TaqManAssay (primer/probe mix), 5 μl diluted cDNA (5 ng), 1.525 μl nuclease‐free water and then loaded into the QuantStudio™ 3D Digital PCR Chip, which has 20,000 mini‐chambers. The reaction conditions were as follows: 96°C for 10 min, 39 cycles at 62°C for 30 s and at 98°C for 30 s, followed by a final extension step at 62°C for 30 s, and then 10°C hold. The data analysis was performed with QuantStudio 3D AnalysisSuite™ Cloud Software version 3.1.2. The quality threshold was set to the default of 0.5, the automatic fluorescence threshold was used, with a 95% confidence level. The final result is expressed as cDNA copies per µl.

### 4.12 Western blot analysis

Whole‐cell lysates were prepared in RIPA buffer containing protease inhibitors, resolved on 10% SDS‐PAGE gels, then electrotransferred onto a PVDF membrane (MilliporeSigma). After blocking with 5% milk in TBST, the membranes were incubated with primary antibodies against CDK4 (1:1000; Cell Signaling Technology), pRb (Ser807/811, 1:1000; Cell Signaling Technology), Rb (1:1000; Cell Signaling Technology), β‐actin (1:1000; Cell Signaling Technology) at 4°C overnight. After incubation with HRP‐conjugated secondary antibody (1:20,000; Abcam) at room temperature for 2 h, the bands were examined using Immobilon Western Chemiluminescent HRP Substrate (MilliporeSigma). Band intensities were quantified with Image J programme.

### 4.13 *In* *vivo* Matrigel plug assay

To assess the angiogenic effects of ciPVT1 *in vivo*, we used a well‐established Matrigel plug assay. Six‐week‐old male BALB/c nude mice were administered a subcutaneous injection of Matrigel (BD Biosciences) supplemented with saline, VEGF, or mixed with HUVECs. After 7 days, the animals were euthanized and the Matrigel plugs were removed, weighed, and photographed. To quantify neovascularization, the hemoglobin concentration in Matrigel homogenates was measured using the QuantiChrom Hemoglobin Assay Kit (DIHB‐250; BioAssay Systems). Formation of microvessels in the plugs was also assessed by CD31 immunofluorescence and hematoxylin and eosin (H&E) staining. Briefly, Matrigel plugs were embedded in OCT compound (Sakura Finetek, 4583) and cut into 5‐μm‐thick sections. After blocking, the sections were incubated with CD31 antibody (Abcam) followed by FITC‐labeled secondary antibodies (Abcam) for 30 min. Images of the sections were examined at a magnification of ×100 under an Invitrogen EVOS FL Auto Cell Imaging System (Thermo Fisher Scientific). Sections with a thickness of 5 μm were also stained with hematoxylin and eosin (H&E). This work was approved by the Animal Care Committee of Guangdong Medical University and adhered to the Guide for the Care and Use of Laboratory Animals published by the US National Institutes of Health (NIH Publication, 8th Edition, 2011).

### 4.14 Study participants

Human arteries were obtained from young (*n* = 7; mean age = 31 years) and old (*n* = 4; mean age = 61 years) individuals at the Affiliated Hospital of Guangdong Medical University who underwent resection of the intestine. Connective tissue surrounding the blood vessels was removed on ice within 2 h after collection. All samples were stored at −80°C until further RNA and protein extraction. Primary criteria for inclusion were the absence of overt life‐threatening diseases such as cancer, serious cardiovascular events, or severe forms of dementia. Informed consent was obtained from all participants. Ethical approval was obtained from the Ethics Committee of the Affiliated Hospital of Guangdong Medical University.

### 4.15 Statistical analysis

At least three independent experiments were conducted for each assay. Data are expressed as mean ± standard deviation (SD), and the difference of data in different groups was investigated using two‐tailed Student's *t* test. Statistical analyses were performed using SPSS Statistics version 20.0 (IBM). A value of *p* ≤ 0.05 was considered to be statistically significant.

## CONFLICT OF INTEREST

The authors declare no competing interests.

## AUTHOR CONTRIBUTIONS

X.M., M.‐Y.C., and X.‐D.X. designed this study. X.M., M.‐Y.C., T.S., Z.‐Y.X., Z.‐F.L., L.‐D.L., W.‐P.W., M.‐Y.Z., and Y.‐L.Z. performed the experiments. X.M. and M.‐Y.C. analyzed the data. X.M. prepared the manuscript. M.‐H.M., S.X., and X.‐G.L. provided suggestions to the study design. X.‐D.X. financed the study and revised the manuscript. All authors approved the final manuscript.

## Supporting information

App S1

## Data Availability

The data that support the findings of this study are available from the corresponding author upon reasonable request.

## References

[acel13529-bib-0001] Barrett, S. P. , & Salzman, J. (2016). Circular RNAs: Analysis, expression and potential functions. Development, 143(11), 1838–1847. 10.1242/dev.128074 27246710 PMC4920157

[acel13529-bib-0002] Barrett, S. P. , Wang, P. L. , & Salzman, J. (2015). Circular RNA biogenesis can proceed through an exon‐containing lariat precursor. Elife, 4, e07540. 10.7554/eLife.07540 26057830 PMC4479058

[acel13529-bib-0003] Boeckel, J.‐N. , Jaé, N. , Heumüller, A. W. , Chen, W. , Boon, R. A. , Stellos, K. , Zeiher, A. M. , John, D. , Uchida, S. , & Dimmeler, S. (2015). Identification and characterization of hypoxia‐regulated endothelial circular RNA. Circulation Research, 117(10), 884–890. 10.1161/CIRCRESAHA.115.306319 26377962

[acel13529-bib-0004] Campisi, J. , & d'Adda di Fagagna, F. (2007). Cellular senescence: When bad things happen to good cells. Nature Reviews Molecular Cell Biology, 8(9), 729–740. 10.1038/nrm2233 17667954

[acel13529-bib-0005] Chen, J. , Li, Y. , Zheng, Q. , Bao, C. , He, J. , Chen, B. , Lyu, D. , Zheng, B. , Xu, Y. , Long, Z. , Zhou, Y. , Zhu, H. , Wang, Y. , He, X. , Shi, Y. , & Huang, S. (2017). Circular RNA profile identifies circPVT1 as a proliferative factor and prognostic marker in gastric cancer. Cancer Letters, 388, 208–219. 10.1016/j.canlet.2016.12.006 27986464

[acel13529-bib-0006] Chen, L. L. (2016). The biogenesis and emerging roles of circular RNAs. Nature Reviews Molecular Cell Biology, 17(4), 205–211. 10.1038/nrm.2015.32 26908011

[acel13529-bib-0007] Cheng, Z. , Yu, C. , Cui, S. , Wang, H. , Jin, H. , Wang, C. , Li, B. , Qin, M. , Yang, C. , He, J. , Zuo, Q. , Wang, S. , Liu, J. , Ye, W. , Lv, Y. , Zhao, F. , Yao, M. , Jiang, L. , & Qin, W. (2019). circTP63 functions as a ceRNA to promote lung squamous cell carcinoma progression by upregulating FOXM1. Nature Communications, 10(1), 3200. 10.1038/s41467-019-11162-4 PMC6642174 31324812

[acel13529-bib-0008] Dellago, H. , Preschitz‐Kammerhofer, B. , Terlecki‐Zaniewicz, L. , Schreiner, C. , Fortschegger, K. , Chang, M. W. F. , Hackl, M. , Monteforte, R. , Kühnel, H. , Schosserer, M. , Gruber, F. , Tschachler, E. , Grillari‐Voglauer, R. , Grillari, J. , Wieser, M. , & Schosserer, M. (2013). High levels of oncomi R‐21 contribute to the senescence‐induced growth arrest in normal human cells and its knock‐down increases the replicative lifespan. Aging Cell, 12(3), 446–458. 23496142 10.1111/acel.12069 PMC3864473

[acel13529-bib-0009] Donato, A. J. , Morgan, R. G. , Walker, A. E. , & Lesniewski, L. A. (2015). Cellular and molecular biology of aging endothelial cells. Journal of Molecular and Cellular Cardiology, 89(Pt B), 122–135. 10.1016/j.yjmcc.2015.01.021 25655936 PMC4522407

[acel13529-bib-0010] Dudekula, D. B. , Panda, A. C. , Grammatikakis, I. , De, S. , Abdelmohsen, K. , & Gorospe, M. (2016). CircInteractome: A web tool for exploring circular RNAs and their interacting proteins and microRNAs. RNA Biology, 13(1), 34–42. 10.1080/15476286.2015.1128065 26669964 PMC4829301

[acel13529-bib-0011] Erusalimsky, J. D. (2009). Vascular endothelial senescence: From mechanisms to pathophysiology. Journal of Applied Physiology (1985), 106(1), 326–332. doi:10.1152/japplphysiol.91353.2008 PMC2636933 19036896

[acel13529-bib-0012] Foreman, K. E. , & Tang, J. (2003). Molecular mechanisms of replicative senescence in endothelial cells. Experimental Gerontology, 38(11–12), 1251–1257. 10.1016/j.exger.2003.09.005 14698804

[acel13529-bib-0013] Herrera, M. D. , Mingorance, C. , Rodriguez‐Rodriguez, R. , & Alvarez de Sotomayor, M. (2010). Endothelial dysfunction and aging: An update. Ageing Research Reviews, 9(2), 142–152. 10.1016/j.arr.2009.07.002 19619671

[acel13529-bib-0014] Hsu, M.‐T. , & Coca‐Prados, M. (1979). Electron microscopic evidence for the circular form of RNA in the cytoplasm of eukaryotic cells. Nature, 280(5720), 339–340. 460409 10.1038/280339a0

[acel13529-bib-0015] Jeck, W. R. , & Sharpless, N. E. (2014). Detecting and characterizing circular RNAs. Nature Biotechnology, 32(5), 453–461. 10.1038/nbt.2890 PMC4121655 24811520

[acel13529-bib-0016] Khee, S. G. , Yusof, Y. A. , & Makpol, S. (2014). Expression of senescence‐associated microRNAs and target genes in cellular aging and modulation by Tocotrienol‐Rich fraction. Oxidative Medicine and Cellular Longevity, 2014, 725929. 10.1155/2014/725929 25132913 PMC4123634

[acel13529-bib-0017] Kong, P. , Yu, Y. , Wang, L. , Dou, Y. Q. , Zhang, X. H. , Cui, Y. , Wang, H. Y. , Yong, Y. T. , Liu, Y. B. , Hu, H. J. , Cui, W. , Sun, S. G. , Li, B. H. , Zhang, F. , & Han, M. (2019). circ‐Sirt1 controls NF‐kappaB activation via sequence‐specific interaction and enhancement of SIRT1 expression by binding to miR‐132/212 in vascular smooth muscle cells. Nucleic Acids Research, 47(7), 3580–3593. 10.1093/nar/gkz141 30820544 PMC6468289

[acel13529-bib-0018] Kristensen, L. S. , Andersen, M. S. , Stagsted, L. V. W. , Ebbesen, K. K. , Hansen, T. B. , & Kjems, J. (2019). The biogenesis, biology and characterization of circular RNAs. Nature Reviews Genetics, 20(11), 675–691. 10.1038/s41576-019-0158-7 31395983

[acel13529-bib-0019] Lal, A. , Navarro, F. , Maher, C. A. , Maliszewski, L. E. , Yan, N. , O'Day, E. , Chowdhury, D. , Dykxhoorn, D. M. , Tsai, P. , Hofmann, O. , Becker, K. G. , Gorospe, M. , Hide, W. , & Lieberman, J. (2009). miR‐24 Inhibits cell proliferation by targeting E2F2, MYC, and other cell‐cycle genes via binding to “seedless” 3'UTR microRNA recognition elements. Molecular Cell, 35(5), 610–625. 10.1016/j.molcel.2009.08.020 19748357 PMC2757794

[acel13529-bib-0020] Li, Z. , Huang, C. , Bao, C. , Chen, L. , Lin, M. , Wang, X. , Zhong, G. , Yu, B. , Hu, W. , Dai, L. , Zhu, P. , Chang, Z. , Wu, Q. , Zhao, Y. , Jia, Y. , Xu, P. , Liu, H. , & Shan, G. (2015). Exon‐intron circular RNAs regulate transcription in the nucleus. Nature Structural & Molecular Biology, 22(3), 256–264. 10.1038/nsmb.2959 25664725

[acel13529-bib-0021] Memczak, S. , Jens, M. , Elefsinioti, A. , Torti, F. , Krueger, J. , Rybak, A. , Maier, L. , Mackowiak, S. D. , Gregersen, L. H. , Munschauer, M. , Loewer, A. , Ziebold, U. , Landthaler, M. , Kocks, C. , le Noble, F. , & Rajewsky, N. (2013). Circular RNAs are a large class of animal RNAs with regulatory potency. Nature, 495(7441), 333–338. 10.1038/nature11928 23446348

[acel13529-bib-0022] Onagoruwa, O. T. , Pal, G. , Ochu, C. , & Ogunwobi, O. O. (2020). Oncogenic role of PVT1 and therapeutic implications. Frontiers in Oncology, 10, 17. 10.3389/fonc.2020.00017 32117705 PMC7010636

[acel13529-bib-0023] Panda, A. C. , Grammatikakis, I. , Kim, K. M. , De, S. , Martindale, J. L. , Munk, R. , Yang, X. , Abdelmohsen, K. , & Gorospe, M. (2017). Identification of senescence‐associated circular RNAs (SAC‐RNAs) reveals senescence suppressor CircPVT1. Nucleic Acids Research, 45(7), 4021–4035. 10.1093/nar/gkw1201 27928058 PMC5397146

[acel13529-bib-0024] Ren, S. , Lin, P. , Wang, J. , Yu, H. , Lv, T. , Sun, L. , & Du, G. (2020). Circular RNAs: Promising molecular biomarkers of human aging‐related diseases via functioning as an miRNA sponge. Molecular Therapy ‐ Methods & Clinical Development, 18, 215–229. 10.1016/j.omtm.2020.05.027 32637451 PMC7326721

[acel13529-bib-0025] Rodier, F. , & Campisi, J. (2011). Four faces of cellular senescence. Journal of Cell Biology, 192(4), 547–556. 10.1083/jcb.201009094 PMC3044123 21321098

[acel13529-bib-0026] Salmena, L. , Poliseno, L. , Tay, Y. , Kats, L. , & Pandolfi, P. P. (2011). A ceRNA hypothesis: The Rosetta Stone of a hidden RNA language? Cell, 146(3), 353–358. 10.1016/j.cell.2011.07.014 21802130 PMC3235919

[acel13529-bib-0027] Salzman, J. , Chen, R. E. , Olsen, M. N. , Wang, P. L. , & Brown, P. O. (2013). Cell‐type specific features of circular RNA expression. PLoS Genetics, 9(9), e1003777. 10.1371/journal.pgen.1003777 24039610 PMC3764148

[acel13529-bib-0028] Tesauro, M. , Mauriello, A. , Rovella, V. , Annicchiarico‐Petruzzelli, M. , Cardillo, C. , Melino, G. , & Di Daniele, N. (2017). Arterial ageing: From endothelial dysfunction to vascular calcification. Journal of Internal Medicine, 281(5), 471–482. 10.1111/joim.12605 28345303

[acel13529-bib-0029] Ungvari, Z. , Tarantini, S. , Kiss, T. , Wren, J. D. , Giles, C. B. , Griffin, C. T. , Murfee, W. L. , Pacher, P. , & Csiszar, A. (2018). Endothelial dysfunction and angiogenesis impairment in the ageing vasculature. Nature Reviews Cardiology, 15(9), 555–565. 10.1038/s41569-018-0030-z 29795441 PMC6612360

[acel13529-bib-0030] Verduci, L. , Ferraiuolo, M. , Sacconi, A. , Ganci, F. , Vitale, J. , Colombo, T. , Paci, P. , Strano, S. , Macino, G. , Rajewsky, N. , & Blandino, G. (2017). The oncogenic role of circPVT1 in head and neck squamous cell carcinoma is mediated through the mutant p53/YAP/TEAD transcription‐competent complex. Genome Biology, 18(1), 237. 10.1186/s13059-017-1368-y 29262850 PMC5738800

[acel13529-bib-0031] Wang, K. , Long, B. , Liu, F. , Wang, J. X. , Liu, C. Y. , Zhao, B. , Zhou, L.‐Y. , Sun, T. , Wang, M. , Yu, T. , Gong, Y. , Liu, J. , Dong, Y.‐H. , Li, N. , & Li, P. F. (2016). A circular RNA protects the heart from pathological hypertrophy and heart failure by targeting miR‐223. European Heart Journal, 37(33), 2602–2611. 10.1093/eurheartj/ehv713 26802132

[acel13529-bib-0032] Wang, P. L. , Bao, Y. , Yee, M.‐C. , Barrett, S. P. , Hogan, G. J. , Olsen, M. N. , Dinneny, J. R. , Brown, P. O. , & Salzman, J. J. P. o. (2014). Circular RNA is expressed across the eukaryotic tree of life. PLoS One, 9(3), e90859. 10.1371/journal.pone.0090859 24609083 PMC3946582

[acel13529-bib-0033] Zhang, X. O. , Wang, H. B. , Zhang, Y. , Lu, X. , Chen, L. L. , & Yang, L. (2014). Complementary sequence‐mediated exon circularization. Cell, 159(1), 134–147. 10.1016/j.cell.2014.09.001 25242744

[acel13529-bib-0034] Zhang, Y. , Zhang, X. O. , Chen, T. , Xiang, J. F. , Yin, Q. F. , Xing, Y. H. , Zhu, S. , Yang, L. , & Chen, L. L. (2013). Circular intronic long noncoding RNAs. Molecular Cell, 51(6), 792–806. 10.1016/j.molcel.2013.08.017 24035497

[acel13529-bib-0035] Zheng, Q. , Bao, C. , Guo, W. , Li, S. , Chen, J. , Chen, B. , Luo, Y. , Lyu, D. , Li, Y. , Shi, G. , Liang, L. , Gu, J. , He, X. , & Huang, S. (2016). Circular RNA profiling reveals an abundant circHIPK3 that regulates cell growth by sponging multiple miRNAs. Nature Communications, 7, 11215. 10.1038/ncomms11215 PMC4823868 27050392

[acel13529-bib-0036] Zhou, M. Y. , Yang, J. M. , & Xiong, X. D. (2018). The emerging landscape of circular RNA in cardiovascular diseases. Journal of Molecular and Cellular Cardiology, 122, 134–139. 10.1016/j.yjmcc.2018.08.012 30118789

