# Supplementary material for: A circular intronic RNA ciPVT1 delays endothelial cell senescence by regulating the miR‐24‐3p/CDK4/pRb axis
Source: Aging Cell. 2021 Dec 13;21(1):e13529. doi: 10.1111/acel.13529 (PMC8761008; doi:10.1111/acel.13529)
Supplement: Supplementary file 1 — App S1 [file ACEL-21-e13529-s001.doc]

**Supplementary methods**

**CircRNAs *in vivo* precipitation (circRIP)**

Biotin-labeled ciPVT1 probe (5′-AAAATCTCTGTTGGCCACCAAAATCCCAG G-3′-biotin) and control probe (5′-TTCTCCGAACGTGTCACGTGACGTGTCAC G-3′-biotin) was designed and synthesized by Guangzhou Epibiotek Co., Ltd. The circRNAs *in vivo* precipitation (circRIP) assay was performed as mentioned previously (Han et al., 2017; Liu et al., 2018). In brief, ciPVT1-overexpressing HUVECs crosslinked by formaldehyde, scraped with lysis buffer, then sonicated and centrifugated. 50 μL of the supernatant was retained as input, and the remaining cell lysis solution was incubated with a ciPVT1-specific probe at 37℃ for 3 h, and then incubated with streptavidin beads at 37℃ for 1 h. The bead-probe-circRNA mixture was washed and incubated with 200 μL of lysis buffer to reverse the formaldehyde crosslinking. Subsequently, RNA was extracted from the mixture using Trizol Reagent. miRNAs were detected by RT-qPCR assay using All-in-One miRNA RT-qPCR Detection Kit (GeneCopoeia, USA).

**Flow cytometric analysis of the cell cycle**

ECs were harvested and fixed with 75% ethanol at 4 °C overnight. After treatment with RNase A at 37 °C for 30 min and staining with propidium iodide, the stained cells were then analyzed by flow cytometry (Becton Dickinson, USA). The results were presented as the percentages of cells in each phase.

**References**

Han, D., Li, J., Wang, H., Su, X., Hou, J., Gu, Y., . . . Cao, X. (2017). Circular RNA circMTO1 acts as the sponge of microRNA-9 to suppress hepatocellular carcinoma progression. *Hepatology, 66*(4), 1151-1164. doi:10.1002/hep.29270

Liu, H., Liu, Y., Bian, Z., Zhang, J., Zhang, R., Chen, X., . . . Zhu, J. (2018). Circular RNA YAP1 inhibits the proliferation and invasion of gastric cancer cells by regulating the miR-367-5p/p27 (Kip1) axis. *Mol Cancer, 17*(1), 151. doi:10.1186/s12943-018-0902-1

**
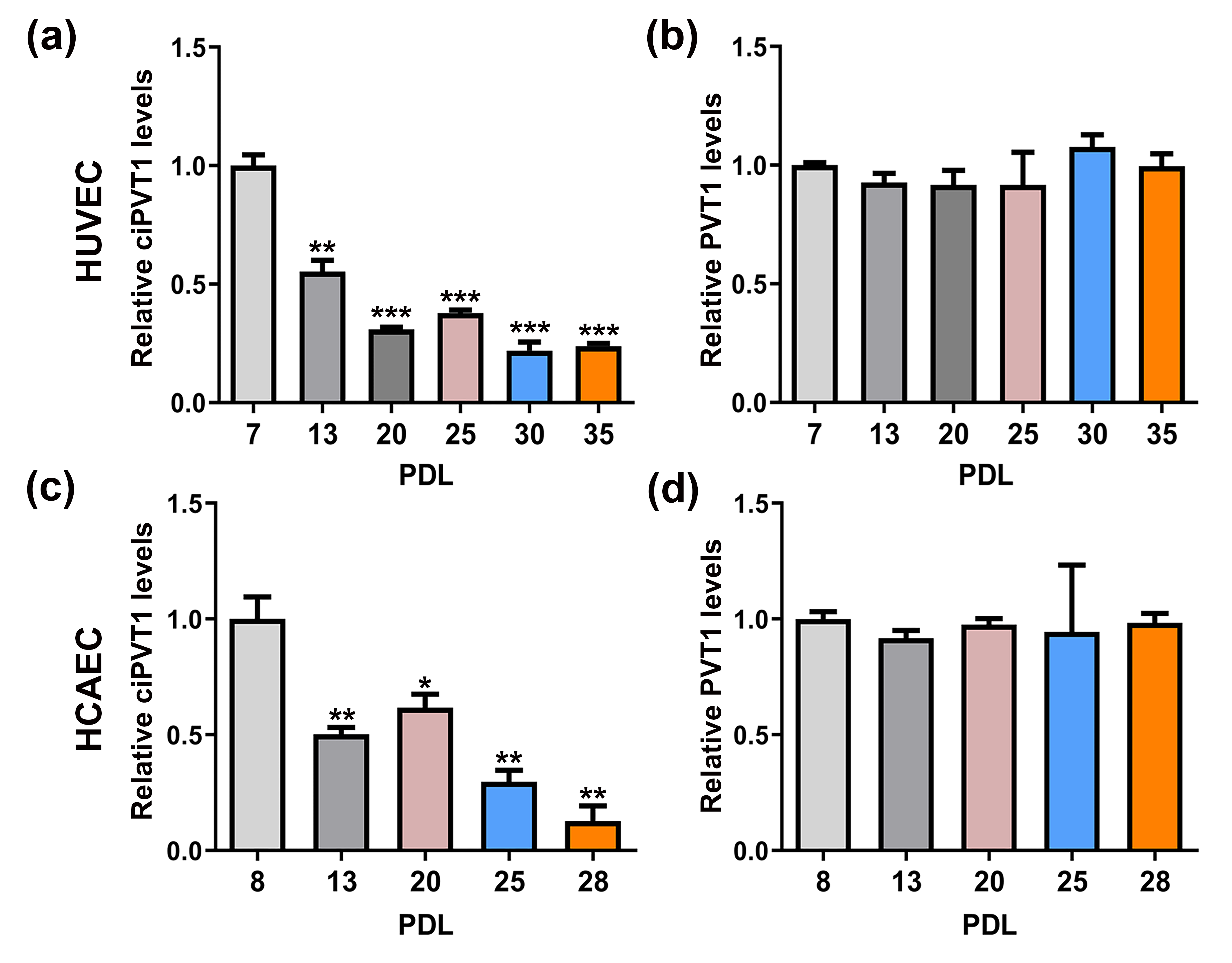
**

**Supplementary Figure S1. Expression of ciPVT1 declined during endothelial cell senescence.** (a,b) RT-qPCR analysis of ciPVT1 and PVT1 RNA at different PDLs of HUVECs. (c,d) RT-qPCR analysis of ciPVT1 and PVT1 RNA at different PDLs of HCAECs. Data are presented as mean ± SD; **P* < 0.05, ***P* < 0.01, ****P* < 0.001.


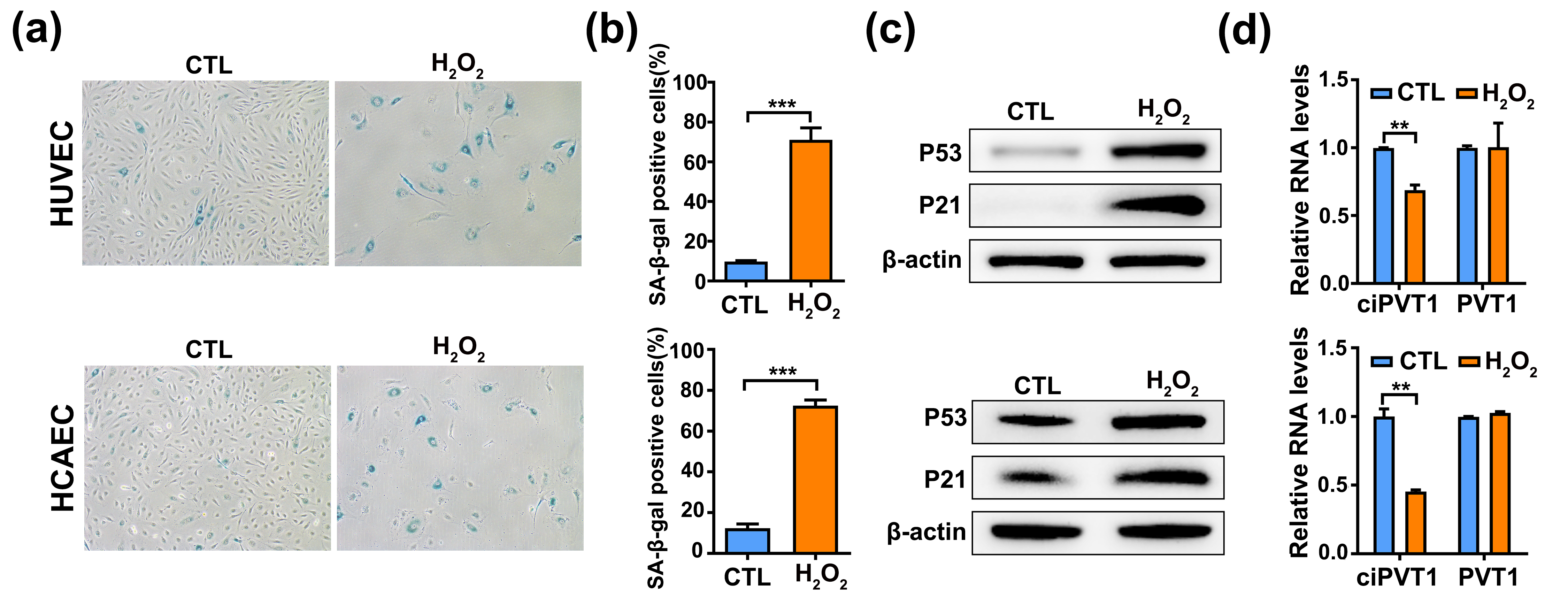


**Supplementary Figure S2.** **Expression of ciPVT1 decreased in H_2_O_2_-induced senescent ECs.** ECs were treated with 100 μmol/L H_2_O_2_ for 1 hour and incubated in complete medium for 7 days. Control (CTL) means untreated cells. (a) Representative photographs of SA-β-gal staining of ECs. (b) The SA-β-gal positive cells were counted and presented as percentage of total cells. (c) Western blot analysis of and P53, P21 and β-actin protein expression in ECs. (d) ciPVT1 and PVT1 RNA expression were quantified by RT-qPCR. Data are presented as mean ± SD; ***P* < 0.01, ****P* < 0.001.

**
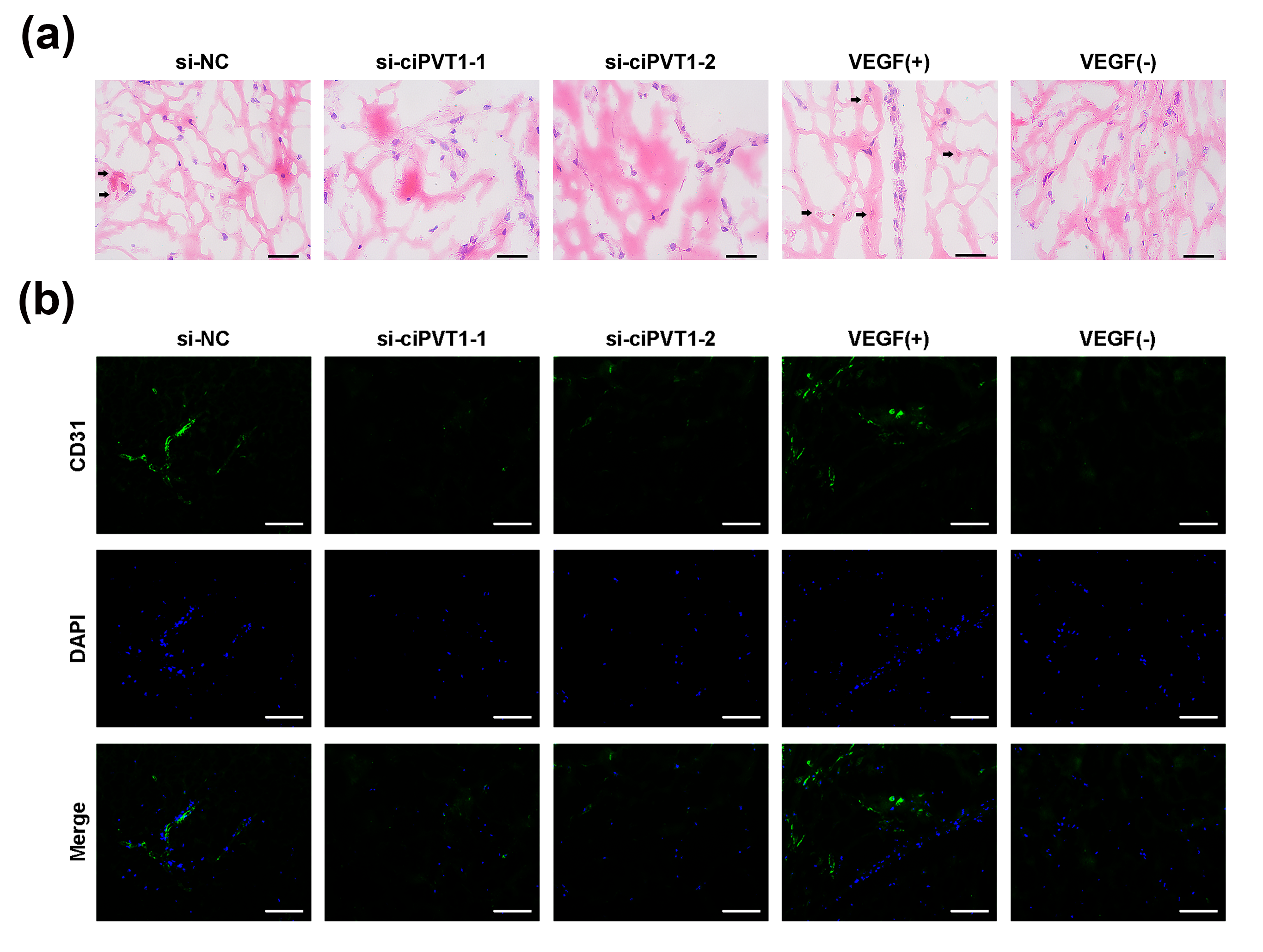
**

**Supplementary Figure S3. Silencing of ciPVT1 decreased angiogenic activity of ECs *in vivo*.** (a) Excised plugs were embedded in OCT compound and stained with H&E to visualize blood vessels. Arrow indicates vessel or capillary-like structure. Scale bar, 50 μm. (b) Plugs were embedded in OCT compound and subjected to immunofluorescence staining for CD31 to visualize blood vessels. Scale bar, 100 μm.

**
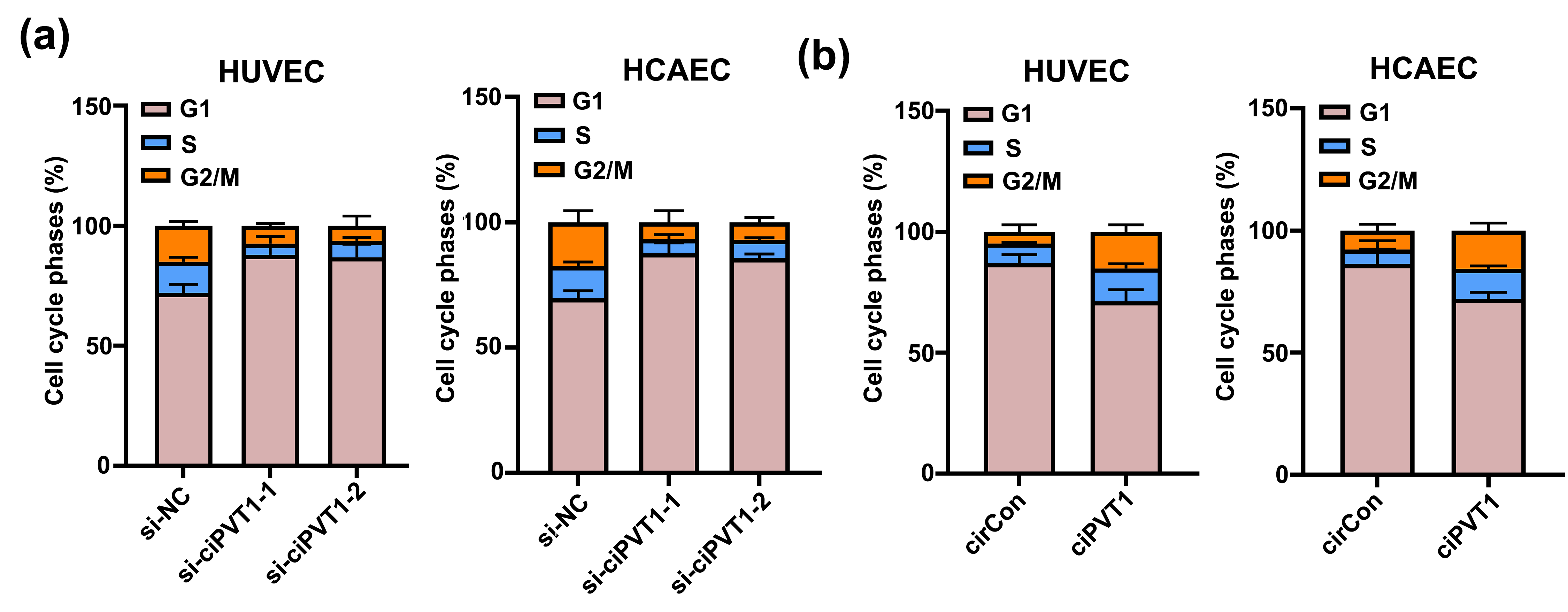
**

**Supplementary Figure S4. ciPVT1 regulated cell cycle progress in ECs.** Flow cytometric cell cycle distribution assays to detect the proportion of ECs in G1, S, and G2/M phases after ciPVT1 inhibition (a) and overexpression (b).


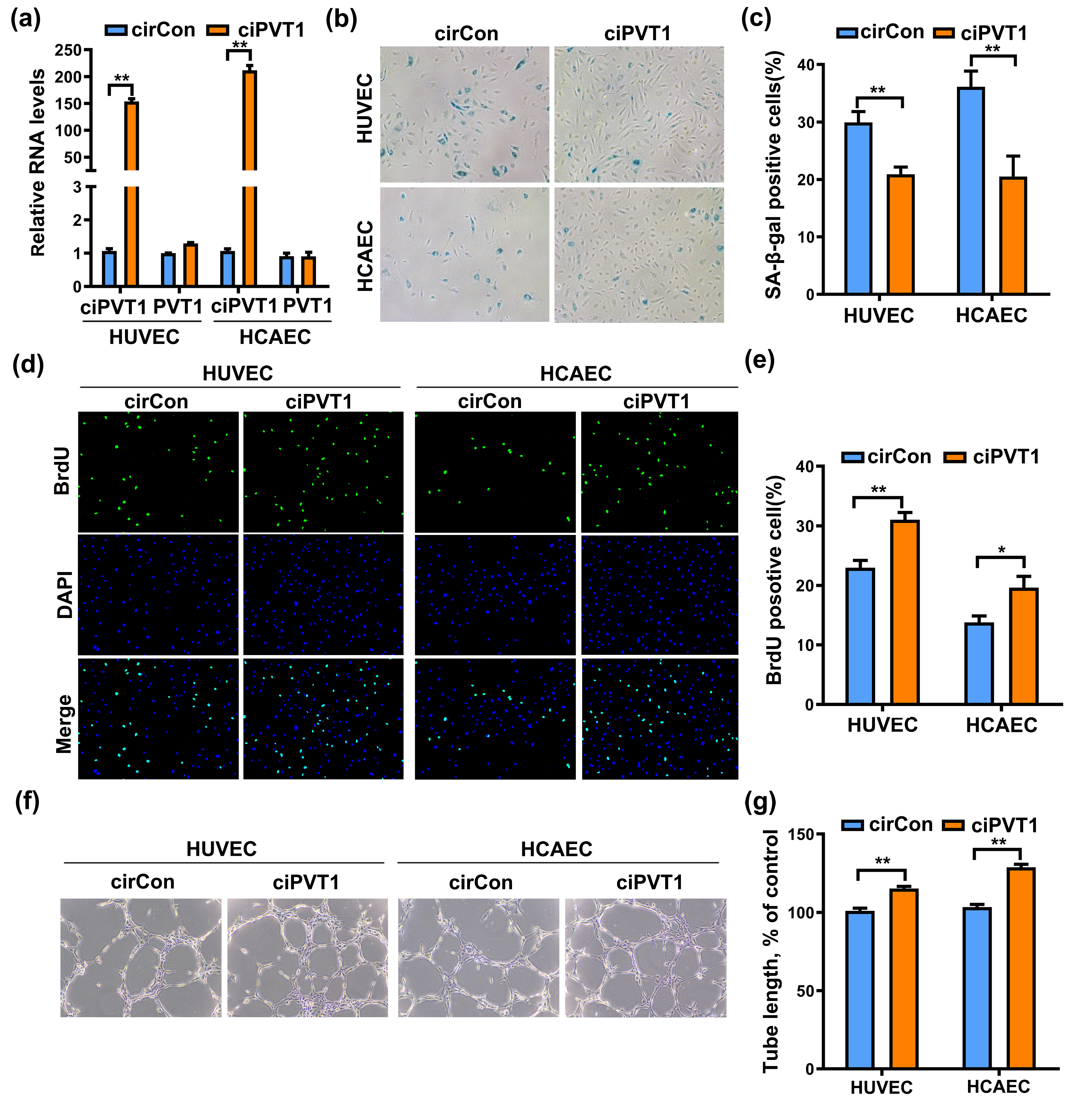


**Supplementary Figure S5. Overexpression of ciPVT1 reduced senescence, promoted proliferation, and increased angiogenic activity of ECs.** (a) Expression of ciPVT1 and PVT1 RNA in ECs infected with the circControl (circCon) or ciPVT1-GFP lentivirus. (b) Representative photographs of SA-β-gal staining of ECs infected with circCon or ciPVT1-GFP lentivirus. (c) The SA-β-gal positive cells were counted and presented as percentage of total cells. (d) Representative images of indicated cells stained for DAPI and BrdU, as a measurement of DNA synthesis in circCon or ciPVT1-GFP lentivirus- infected ECs. (e) The BrdU positive cells were counted and presented as percentage of total cells. (f, g) Representative micrographs and statistical summary of *in vitro* Matrigel assays in circCon or ciPVT1-GFP lentivirus- infected ECs. Data are presented as mean ± SD; **P* < 0.05, ***P* < 0.01.

**
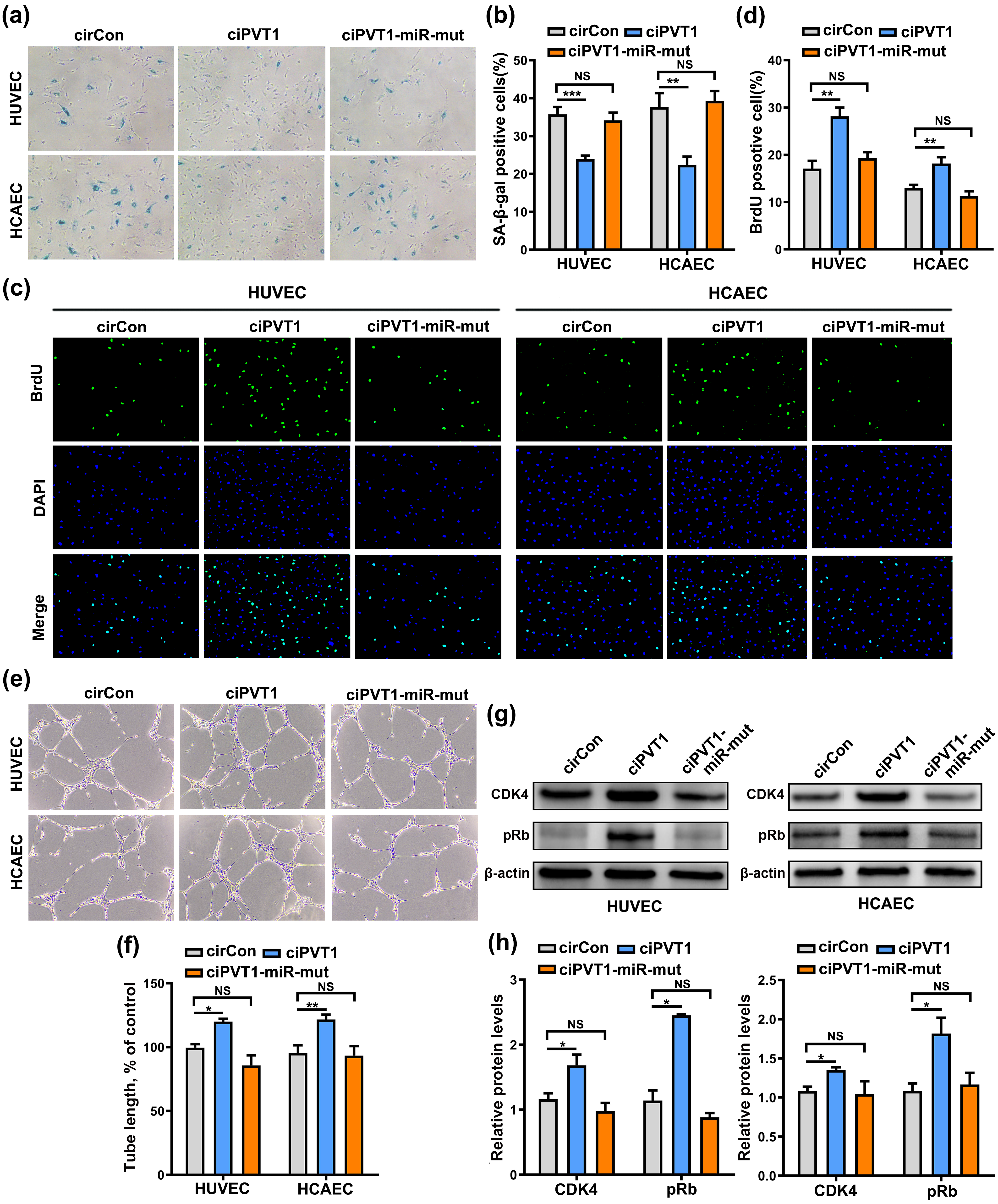
**

**Supplementary Figure S6. Overexpression of ciPVT1-miR-mut had no effect on cellular senescence, proliferation, angiogenic activity of ECs.** (a) Representative photographs of SA-β-gal staining of ECs infected with circCon, ciPVT1- or ciPVT1-miR-mut-GFP lentivirus. (b) The SA-β-gal positive cells were counted and presented as percentage of total cells. (c) Representative images of indicated cells stained for DAPI and BrdU, as a measurement of DNA synthesis in ECs infected with circCon, ciPVT1- or ciPVT1-miR-mut-GFP lentivirus. (d) The BrdU positive cells were counted and presented as percentage of total cells. (e,f) Representative micrographs and statistical summary of *in vitro* Matrigel assays in ECs infected with circCon, ciPVT1- or ciPVT1-miR-mut-GFP lentivirus. (g,h) CDK4, pRb protein expression and intensity ratio between CDK4, pRb, and β-actin in ECs infected with circCon, ciPVT1- or ciPVT1-miR-mut-GFP lentivirus. Data are presented as mean ± SD; **P* < 0.05, ***P* < 0.01, ****P* < 0.001. NS means no significant difference.

**
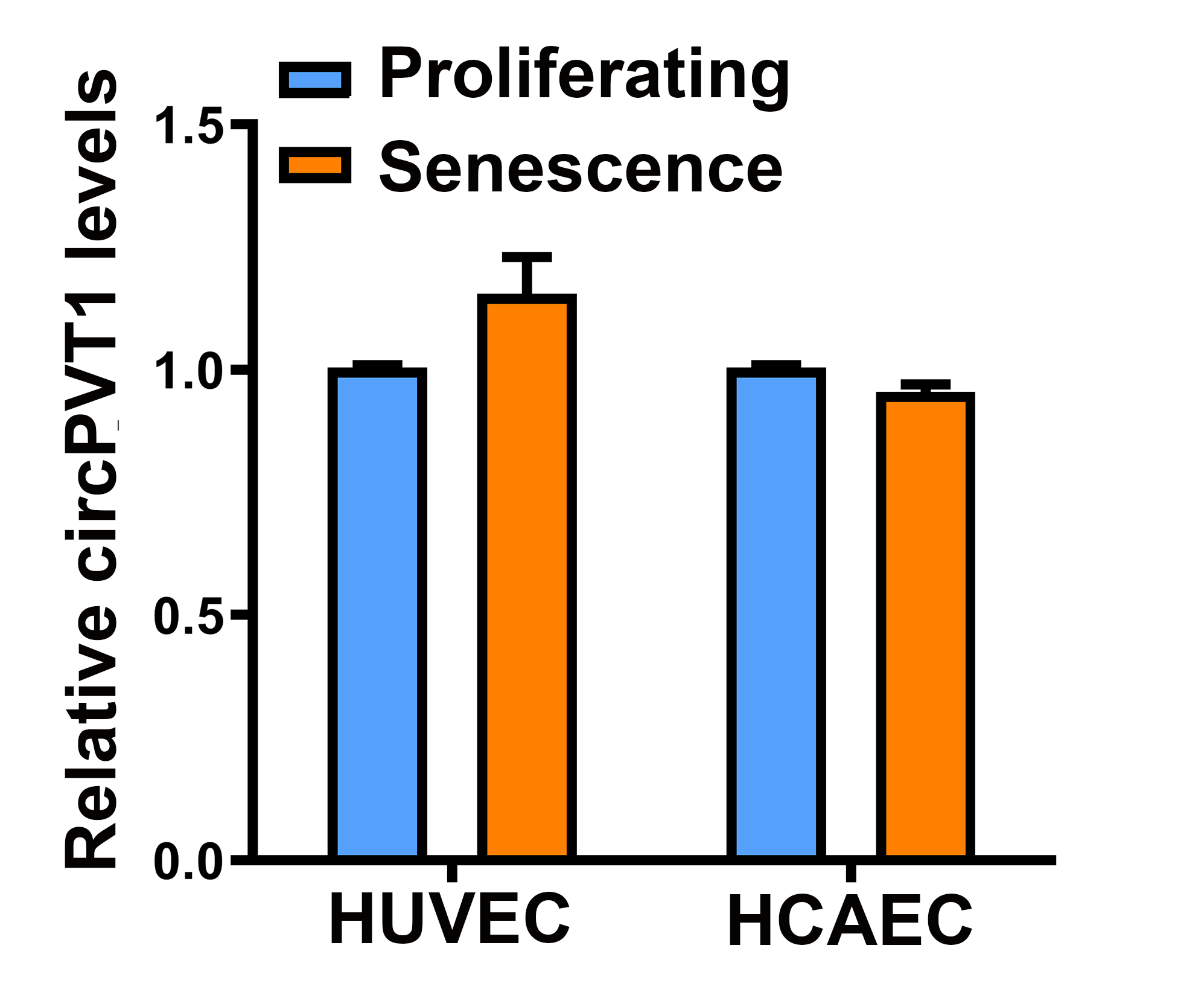
**

**Supplementary Figure S7. CircPVT1 expression in proliferating and senescent ECs.** RT-qPCR analysis of circPVT1 levels in proliferating and senescent ECs. Data are presented as mean ± SD.

Supplementary Table S1 Identification of ciPVT1-associated miRNAs by circRIP

| **miRNA ID** | **C_t_** | | **2^-ΔCt^** | | **Fold Difference** |
| --- | --- | --- | --- | --- | --- |
|  | **ciPVT1** | **Control** | **ciPVT1** | **Control** | **ciPVT1/Control** |
| **miR-24-3p** | **26.17** | **35.82** | **1.3E-08** | **1.6E-11** | **803.41** |
| miR-140-3p | 23.93 | 33.17 | 6.3E-08 | 1.0E-10 | 604.67 |
| miR-146b-5p | 27.25 | 35.32 | 6.3E-09 | 2.3E-11 | 268.73 |
| miR-140-5p | 30.6 | 38.28 | 6.1E-10 | 3.0E-12 | 205.07 |
| miR-302b-3p | 30.5 | 37.83 | 6.6E-10 | 4.1E-12 | 160.90 |
| miR-331-3p | 30.91 | 38.22 | 5.0E-10 | 3.1E-12 | 158.68 |
| miR-22-3p | 26.26 | 32.45 | 1.2E-08 | 1.7E-10 | 73.01 |
| miR-142-3p | 34.5 | 40 | 4.1E-11 | 9.1E-13 | 45.25 |
| miR-103a-3p | 26.18 | 31.2 | 1.3E-08 | 4.1E-10 | 32.45 |
| miR-96-5p | 30.41 | 35.13 | 7.0E-10 | 2.7E-11 | 26.35 |
| miR-19b-3p | 27.81 | 32.44 | 4.2E-09 | 1.7E-10 | 24.76 |
| miR-15a-5p | 27.21 | 31.66 | 6.4E-09 | 2.9E-10 | 21.86 |
| miR-200a-3p | 27.46 | 31.84 | 5.4E-09 | 2.6E-10 | 20.82 |
| miR-181a-5p | 24.46 | 28.78 | 4.3E-08 | 2.2E-09 | 19.97 |
| miR-27a-3p | 24.43 | 28.74 | 4.4E-08 | 2.2E-09 | 19.84 |
| miR-101-3p | 28.94 | 33.25 | 1.9E-09 | 9.8E-11 | 19.84 |
| miR-584-5p | 25.68 | 29.99 | 1.9E-08 | 9.4E-10 | 19.84 |
| miR-222-3p | 26.45 | 30.68 | 1.1E-08 | 5.8E-10 | 18.77 |
| miR-18b-5p | 30.7 | 34.91 | 5.7E-10 | 3.1E-11 | 18.51 |
| miR-486-5p | 29.9 | 34.1 | 1.0E-09 | 5.4E-11 | 18.38 |
| miR-27b-3p | 24.64 | 28.83 | 3.8E-08 | 2.1E-09 | 18.25 |
| miR-181c-5p | 23.8 | 27.96 | 6.8E-08 | 3.8E-09 | 17.88 |
| miR-221-3p | 27.46 | 31.62 | 5.4E-09 | 3.0E-10 | 17.88 |
| miR-29c-3p | 24.94 | 29.1 | 3.1E-08 | 1.7E-09 | 17.88 |
| miR-199a-3p | 32.11 | 36.19 | 2.2E-10 | 1.3E-11 | 16.91 |
| miR-708-5p | 25.33 | 29.38 | 2.4E-08 | 1.4E-09 | 16.56 |
| miR-34b-5p | 30.68 | 34.71 | 5.8E-10 | 3.6E-11 | 16.34 |
| miR-30d-5p | 25.83 | 29.8 | 1.7E-08 | 1.1E-09 | 15.67 |
| miR-302d-3p | 28.71 | 32.64 | 2.3E-09 | 1.5E-10 | 15.24 |
| miR-30a-5p | 25.41 | 29.33 | 2.2E-08 | 1.5E-09 | 15.14 |
| miR-365a-5p | 25.48 | 29.39 | 2.1E-08 | 1.4E-09 | 15.03 |
| miR-30b-5p | 25.82 | 29.72 | 1.7E-08 | 1.1E-09 | 14.93 |

**Supplementary Table S2** Oligomers used in this study

| **Oligomer Name** | **Sequence (5’-3’)** | |
| --- | --- | --- |
| β-actin-F (convergent) | | CATGTACGTTGCTATCCAGGC |
| β-actin-R (convergent) | | CTCCTTAATGTCACGCACGAT |
| β-actin-F (divergent) | | AAATCGTGCGTGACATTAAGGAGA |
| β-actin-R (divergent) | | CATACCCCTCGTAGATGGGCA |
| ciPVT1-F (divergent) | | AAGACCTGGGATTTTGGTGG |
| ciPVT1-R (divergent)  ciPVT1-F (convergent)  ciPVT1-R (convergent)  PVT1-F  PVT1-R | | TCAGCACTGGCACAACCACT  TATGGCTCCACCCAGAAG  AGCTCATAGGTTAGGGATTTT  CTTCCAGTGGATTTCCTTGC  CATCTTGAGGGGCATCTTTT |
| circPVT1-F | | CGACTCTTCCTGGTGAAGCATCTGAT |
| circPVT1-R | | TACTTGAACGAAGCTCCATGCAGC |
| si-NC  si-ciPVT1-1  si-ciPVT1-2  ciPVT1 FISH probe  miR-24-3p FISH probe | | UUCUCCGAACGUGUCACGUTT  CTGGGATTTTGGTGGCCAA  GGTGGCCAACAGAGATTTT  TGTTGGCCACCAAAATCCCAGGTCTTGAT  CTGTTCCTGCTGAACTGAGCCA |
